# An immune responsive tumor microenvironment imprints into PBMCs and predicts outcome in advanced pancreatic cancer: lessons from the PREDICT trial

**DOI:** 10.1186/s12943-025-02406-7

**Published:** 2025-07-22

**Authors:** Anton Lahusen, Manfred P. Lutz, Rui Fang, Martina Kirchner, Sarah Albus, Klaus Kluck, Meinolf Karthaus, Andreas Schwarzer, Gabriele Siegler, Alexander Kleger, Thomas J. Ettrich, Alexander Becher, Sabine Höfling, Jens T. Siveke, Jan Budczies, Andrea Tannapfel, Albrecht Stenzinger, Phyllis Fung-Yi Cheung, Tim Eiseler, Thomas Seufferlein

**Affiliations:** 1 Department of Internal Medicine I, Ulm University Hospital, Albert-Einstein-Allee 23, 89081 Ulm, Germany; 2 Department of Gastroenterology, Caritas Klinikum Saarbrücken St. Theresia, Saarbrücken, Germany; 3 Bridge Institute of Experimental Tumor Therapy (BIT), West German Cancer Center, University Hospital Essen, University of Duisburg-Essen, Essen, Germany; 4 Division of Solid Tumor Translational Oncology, German Cancer Consortium (DKTK), Partner Site Essen, a partnership between German Cancer Research Center (DKFZ) and University Hospital Essen, Essen, Germany; 5 Institute of Pathology, Heidelberg University Hospital, Heidelberg, Germany; 6 Center for Personalized Medicine (ZPM), Heidelberg University Hospital, Heidelberg, Germany; 7 Translational Lung Research Center (TLRC), Member of the German Center for Lung Research (DZL), Heidelberg University Hospital, Heidelberg, Germany; 8 Institute of Pathology, Ruhr University Bochum, Bochum, Germany; 9 Department of Hematology, Oncology, and Palliative Care, München Klinik Harlaching and Neuperlach, München, Germany; 10 Onkopraxis Probstheida, Leipzig, Germany; 11 Hämatologie/Onkologie, Klinikum Nürnberg Nord/Paracelsus Medizinische Privatuniversität, Nürnberg, Germany; 12 Institute for Molecular Oncology and Stem Cell Biology, Ulm University Hospital, Ulm, Germany; 13 Division of Interdisciplinary Pancreatology, Department of Internal Medicine I, Ulm University Hospital, Ulm, Germany; 14 CROLLL GmbH, Nürnberg, Germany; 15 National Center for Tumor Diseases (NCT) West, Campus Essen, Essen, Germany; 16 Spatiotemporal Tumor Heterogeneity, DKTK, Partner Site Essen, A partnership between DKFZ and University Hospital Essen, Essen, Germany

**Keywords:** Advanced pancreatic ductal adenocarcinoma, Tumor immune microenvironment, Peripheral blood mononuclear cells, Machine learning, Liquid biomarkers, Immunophenotyping, Second-line chemotherapy

## Abstract

**Background:**

Prognosis in advanced pancreatic ductal adenocarcinoma (aPDAC) is particularly poor, only few patients benefit from treatment, and there are few biomarkers. The PREDICT trial examined whether first-line time-to-treatment failure (TTF1) predicts second-line treatment failure (TTF2) in aPDAC patients but found no association. We hypothesized that the tumor immune microenvironment (TiME) could correlate with the outcome in this trial and assessed whether tissue features were reflected in peripheral blood.

**Methods:**

PREDICT patients received 5-FU/LV plus nanoliposomal irinotecan as second-line treatment. We stratified patients by shortest vs. longest TTF2 and analyzed 20 treatment-naïve tumor tissues samples via transcriptomics and immunohistochemistry. Peripheral blood mononuclear cells (PBMCs) from 82 patients collected prior to second-line therapy underwent flow cytometry and gene expression profiling. A machine learning pipeline integrated PBMC and clinical data to predict second-line outcome including external validation in 30 patients.

**Results:**

Long-TTF2 tumors exhibited an immune-active (“hot”) TiME with cytotoxic CXCR3^+^CD8^+^-T-cell infiltration. PBMC analysis showed that these immune features were reflected in peripheral blood after one line of treatment. A novel 7-feature PBMC-based model (“TTF2Pred”) accurately predicted TTF2 and overall survival, outperforming clinical or CA19-9 models and was confirmed in an external validation cohort. Long-TTF2 patients exhibited more circulating CXCR3⁺-T-cells and plasmacytoid dendritic cells. Short-TTF2 patients had more platelet-leukocyte aggregates.

**Conclusions:**

An immune-active, treatment-naïve TiME predicts a better second-line outcome, and these characteristics imprinted into PBMCs obtained after one line of chemotherapy. We here first describe a minimally invasive, PBMC-based predictor of second-line outcome as a powerful prognostic tool for triaging patients.

**Trial Registration:**

ClinicalTrials.gov NCT03468335 (registered March 15, 2018).

**Supplementary Information:**

The online version contains supplementary material available at 10.1186/s12943-025-02406-7.

## Background

Pancreatic ductal adenocarcinoma (PDAC) has a dismal prognosis with a five-year overall survival rate of only 13% [1]. Most patients present with metastatic disease at diagnosis [2]. Although systemic chemotherapy (CTX) extends survival, median overall survival in the metastatic situation rarely exceeds one year [3]. After failure of first-line treatment, nanoliposomal irinotecan (Nal-IRI) plus 5-Fluorouracil (5-FU)/leucovorin (LV) showed some improvement in clinical outcome and is the only internationally approved second-line regimen [4]. However, only a subset of patients benefits and predictive biomarkers for advanced PDAC (aPDAC) remain scarce [4–6]. The PREDICT trial in patients with aPDAC examined prospectively whether the time-to-treatment failure in first-line (TTF1) with gemcitabine/nab-paclitaxel (Gem-nabPac) would predict second-line outcome (TTF2) with Nal-IRI/5-FU/LV, but there was no association [7]. The NAPOLI1 trial further indicated that second-line outcome for this treatment can vary widely [4], highlighting the need for individualized treatment decisions in second-line therapy and for robust biomarkers to guide such decisions.

Biomarker research in PDAC is largely focused on primary tumor tissues. Transcriptomic profiling has identified prognostic signatures [8, 9] such as the Moffitt subtypes [10] and GemPred+ for gemcitabine sensitivity [11]. Implementation of such signatures in clinical practice is still challenging, and data in the advanced setting is limited. Restrictions result from treatment-induced tumor evolution, the invasive repeated biopsies, putative sampling bias due to intratumoral heterogeneity, and difficulties in obtaining sufficient tissue [12–14]. Liquid biopsies may overcome many of these limitations and are increasingly explored for biomarker discovery, particularly for residual disease monitoring and treatment response prediction in the advanced setting [12–15]. Carbohydrate antigen 19-9 (CA19-9) is currently the only clinically established liquid biomarker for PDAC used for diagnosis, prognostication, and disease monitoring [13, 16]. However, its clinical utility is limited in ~10% of individuals with a Lewis antigen-negative blood type and due to non-specific elevation in PDAC comorbidities such as obstructive jaundice and pancreatitis [16, 17]. Thus, multi-analyte blood-based signatures integrated by computational approaches are increasingly investigated [12, 14] focusing on e.g., circulating tumor DNA, proteins, or exosomes [18]. Peripheral blood mononuclear cells (PBMCs) represent a so-far less explored source of predictive liquid biomarkers in PDAC. A small number of studies have linked PBMC subsets to PDAC differential diagnosis and prognosis [19–21], including e.g., a diagnostic 8-gene PBMC panel [19]. Prognostic or predictive signatures may not only arise from the tumor itself, but also from the tumor (immune) microenvironment (TME, TiME). The PDAC TiME is predominantly immunosuppressive, marked by a dense desmoplastic stroma that impedes effector T‐cell infiltration and fosters the accumulation of e.g., regulatory T-cells [22, 23]. Recent multimodal analysis of PDAC TiME and matched blood samples revealed a complex immune network, with dysfunction in both tumor and peripheral blood [24]. In addition, the TiME evolves in response to cancer therapies with distinct features that predict clinical outcome [25–27].

We hypothesized that a more favorable TiME in treatment-naïve tumors could correlate with a better outcome of patients with aPDAC in the PREDICT trial. Thus, we analyzed whether PBMCs obtained prior to second-line treatment may reflect features from treatment-naïve tumor tissues, providing reproducible, minimally invasive markers with predictive value for patient outcome. These features may reflect therapy-naïve TiME imprinting. However, we cannot exclude chemotherapy also induced remodeling to some degree. Such an immune signature could guide treatment decisions for aPDAC patients with poor prognosis not benefitting from standard treatment.

## Methods

### Patient cohorts and study design

**Fig. 1:**
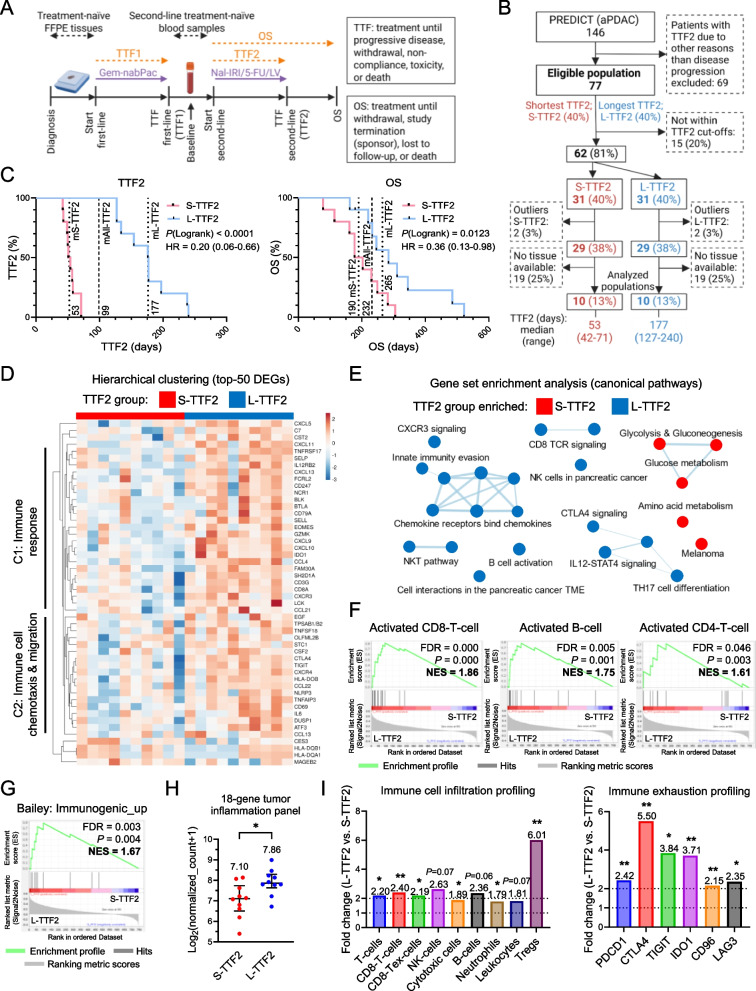
Immune-enriched tumor microenvironment in treatment-naïve PDAC tumors predicts extended second-line chemotherapy benefit. **A** Overview of the PREDICT trial design. FFPE tumor tissues were collected at diagnosis (treatment-naïve), and PBMCs were obtained immediately prior to second-line therapy. TTF: Time-to-treatment failure; OS: Overall survival. **B** Workflow for PREDICT tissue cohort selection. Patients with aPDAC and TTF2 due to disease progression were ranked by TTF2, selecting the top and bottom 40^th^-percentiles. Potential outliers and cases lacking tissue samples were excluded, yielding 10 patients per group (S-/L-TTF2). **C** Kaplan-Meier survival curves for TTF2 and OS in S-/L-TTF2 groups. Log-rank *P*-values, hazard ratios (HR) with 95% confidence interval, and median TTF2/OS values (per cohort and for the combined group) are shown. **D** Hierarchical clustering of the top-50 differentially expressed genes (DEGs) from NanoString analysis. Rows were clustered using correlation distance and average linkage, columns by binary distance and average linkage. Gene clusters (C1, C2) were functionally annotated using GO biological process (BP) terms via overrepresentation analysis. **E** Gene set enrichment analysis (GSEA) radial heatmap displaying enriched cellular pathways (Molecular Signatures Database, MSigDB: WikiPathways, WP; Gene Ontology, GO; Pathway Interaction Database, PID; BioCarta; Reactome). Node size represents normalized enrichment scores (NES). Similarity/q-value cut-offs: 0.5/0.2 (*P* < 0.05). **F** GSEA-based immune cell subset profiling (FDR < 0.05) using TISIDB gene signatures. **G** GSEA of PdacR PDAC subclass signatures enriched in L-TTF2 group tumors (FDR < 0.05). **H** Mean relative RNA expression of a NanoString 18-gene tumor-inflammation signature in L-/S-TTF2 tumors. **I** Fold change in mean relative RNA expression of NanoString immune cell gene signatures (*P* ≤ 0.07) and immune checkpoint/exhaustion markers (*P* < 0.05) in L-/S-TTF2 tumors. Schemes were generated using BioRender. *P ≤ 0.05, **P ≤ 0.01

The PREDICT trial (AIO-PAK-0216; EudraCT: 2016-005147-17; ClinicalTrials.gov: NCT03468335, https://clinicaltrials.gov/study/NCT03468335, accessed 15 April 2025) was a multicenter, open-label, single-arm phase IIIb study conducted across 40 German centers (March 2018-May 2022). Of 152 enrolled patients, 146 received treatment. Eligible patients (≥ 18 years) had confirmed aPDAC following first-line Gem-nabPac failure and required second-line therapy. Key inclusion criteria were ECOG performance status of 0-2 and adequate organ function. Patients with conditions affecting treatment evaluation or safety were excluded. The study was ethics-approved, and all patients provided written informed consent, including for translational research participation. Clinical characteristics, including TTF1, were collected both at diagnosis and prior to the start of second-line treatment (study baseline; Fig. 1A). The second-line regimen included nanoliposomal irinotecan (Nal-IRI) 70 mg/m^2^ (1.5-h infusion), 5-fluorouracil (5-FU) 2400 mg/m^2^ (46-h infusion), and leucovorin (LV) 400 mg/m^2^ (0.5-h infusion) on day 1 of each 2-week cycle (q2w). Treatment continued until progression, withdrawal, non-compliance, toxicity, or death (TTF2). Outcome further included overall survival (OS) defined from the start of second-line therapy to withdrawal, sponsor termination, loss to follow-up, or death (Fig. 1A).

FFPE tumor tissues (1 block/patient) were collected at diagnosis before first-line therapy (Fig. 1A). PBMCs (1 EDTA vial/patient) were isolated immediately prior to second-line CTX (Fig. 1A). Our study using the PREDICT patient cohort employed an extreme phenotype design, selecting patients from the top and bottom 40^th^-45^th^ percentiles of treatment response distributions in the “tissue” and “PBMC” cohorts. We performed NanoString IO360 or flow cytometry profiling of tumor samples and PBMCs, respectively, to identify markers that may explain the striking differences in patient outcome upon second-line Nal-IRI/5-FU/LV therapy. This enrichment strategy was essential for identifying robust biological differences but inherently also captured patients with divergent clinical characteristics. To evaluate whether the identified immune signatures provided predictive and prognostic value beyond these clinical differences, we employed comparative machine learning approaches and external validation in cohorts with different clinical characteristics. Consequently, we identified the TTF2Pred minimal predictive model for triaging patients according to second-line therapy outcome before second-line therapy onset.

Besides Nanostring profiling, transcriptomic analysis also included publicly available datasets: TCGA-PAAD (RNA-seq; *n* = 179; https://portal.gdc.cancer.gov/projects/TCGA-PAAD, accessed 15 April 2025) [28] and GSE154778 (single-cell RNA-seq; *n* = 16; https://www.ncbi.nlm.nih.gov/geo/query/acc.cgi?acc=GSE154778, accessed 15 April 2025) [29]. The external validation cohort (eV-Set) obtained from the University Hospital Ulm/Department of Internal Medicine biobank (*n* = 30 patients; February 2014-July 2022) included aPDAC patients with documented TTF1, TTF2, and OS who received variable second-line regimens (Fig. 5G). PBMCs were collected directly before second-line CTX. Ethics approval and informed consent from all patients prior to inclusion into the biobank were obtained.

### Tissue transcriptomics, multiplexed immunohistochemistry, feature acquisition pipeline, machine learning, and Wright-Giemsa staining

Details available in supplementary methods and Table S1.

### Statistical analysis

Statistical analysis used GraphPad Prism v10.4.1 (RRID:SCR_002798), R v4.3.3 (RRID:SCR_001905), Microsoft Excel v16.0 (RRID:SCR_016137), GSEA_4.3.2 (RRID:SCR_003199) [30], ClustVis (RRID:SCR_017133) [31], Cytoscape 3.10.3 (RRID:SCR_003032) [32], GEPIA2 (RRID:SCR_026154) [33], TISIDB (RRID:SCR_018821) [34], ShinyGO (RRID:SCR_019213) [35], TISCH2 (RRID:SCR_018821) [36], STITCH (RRID:SCR_007947) [37], and XLSTAT v2024.4 (RRID:SCR_016299). Kaplan–Meier curves were analyzed via log-rank tests. Cox regression used exact estimation (Efron’s approximation for larger ties). Outliers were removed by ROUT (Q = 2%). Normality was assessed by D’Agostino-Pearson testing. Dichotomous comparisons used unpaired t-test or Mann-Whitney-U test. Multiple comparisons were analyzed via multiple unpaired t-tests or one-way ANOVA with Sidak’s post-hoc test. Paired data were assessed using the Wilcoxon signed-rank test. Categorical variables used Fisher’s exact test. Correlations used the Spearman method. Significance was set at *P* < 0.05 (adjusted for multiple testing where applicable).

## Results

### Immune-enriched and T-cell-inflamed “hot” tumor microenvironment in treatment-naïve PDAC tumors of long-TTF2 patients

The first objective of this study was to identify differences in the TiME of treatment-naïve PDAC tissues from patients of the PREDICT trial that clearly benefited from a second-line CTX with Nal-IRI/5-FU/LV as opposed to patients with poor outcome. These data were used to inform about molecular and biological alterations that could guide further identification of biomarkers for second-line outcome, including liquid biomarkers.

Therapy-naïve PDAC tumor specimens (resections and biopsies; Table S2) of 10 patients per group from the PREDICT trial were evaluated representing either short (S-TTF2) or long (L-TTF2) second-line treatment durations, i.e., the bottom and top 40^th^-percentiles of TTF2 values, respectively (Fig. S1A, Fig. 1A-B). This selection was made to allow maximum separation, but also a sensible number of patients per group and is supported by a priori statistical power estimation (supplementary methods). Clinical and sample characteristics showed that L-TTF2 patients had fewer liver metastases (diagnosis, prior to the start of second-line therapy), and S-TTF2 patients had higher CA19-9 prior to the start of second-line therapy, albeit with a broad range (Fig. 1A, Table S2). All other parameters were comparable (Table S2). By design, median TTF2 from the start of second-line therapy was substantially longer in the L-TTF2 group (Fig. 1B-C; mTTF2: 177 vs. 53 days, *P* < 0.0001). TTF2 correlated positively with second-line CTX cycles and overall survival (OS) from the start of second-line therapy, but not with TTF1 (Fig. S1B; Table S2).

To explore molecular and biological differences in the TiME of the respective tumors prior to any treatment, a NanoString transcriptional tumor profiling (IO360 panel) revealed substantial differences in the immunological profile between the groups (Table S3). Clustering of differentially expressed genes (DEGs) distinguished L-TTF2 tumors by upregulated immune response and migration genes (Fig. S2A, Fig. 1D). Overrepresentation analysis (ORA) confirmed that immune response and cell migration pathways were enriched the most (Fig. S2B), and principal component analysis (PCA) also segregated the two groups (Fig. S2C).

Gene set enrichment analysis (GSEA) further indicated an immune-active, T-cell-inflamed TiME in L-TTF2 patients (Fig. 1E). L-TTF2 tumors showed enrichment of multiple immune signaling pathways including CXCR3, CD8⁺-T-cell receptor, NK-cell interactions, and lymphocyte activation (Fig. 1E-F; Fig. S2D, Table S4). Consistently, a *Bailey immunogenic_up* gene signature (NES = 1.67, *P* = 0.004) associated with long-term survival [8] and a NanoString tumor-inflammation 18-gene signature (*P* = 0.016) were enriched in L-TTF2 tumors (Fig. 1G-H). In contrast, S-TTF2 tumors were enriched for a *Collison quasi-mesenchymal* subtype linked to poor prognosis [9] (NES = –1.73, *P* = 0.029; Fig. S2E; Table S4). The NanoString tumor panel (Table S3-4) detected in treatment-naïve tumors of the L-TTF2 group higher immune infiltration (e.g., cytotoxic T-cells, B-cells) and checkpoint markers (e.g., *PDCD1, CTLA4, TIGIT*; Fig. 1I, Fig. S2F)*.* This indicated an active immune response under adaptive regulation. Overall, treatment-naïve tumors from L-TTF2 patients exhibited a distinct “hot” TiME characterized by T-cell infiltration and activation. These therapy-naive immune features appear to predispose for a more positive outcome in the advanced setting.

### Activated CXCR3/CD8-T-cells recruited by CXCL9/10/11/13 chemokines in treatment-naïve PDAC distinguish long-TTF2 from short-TTF2 patients

**Fig. 2:**
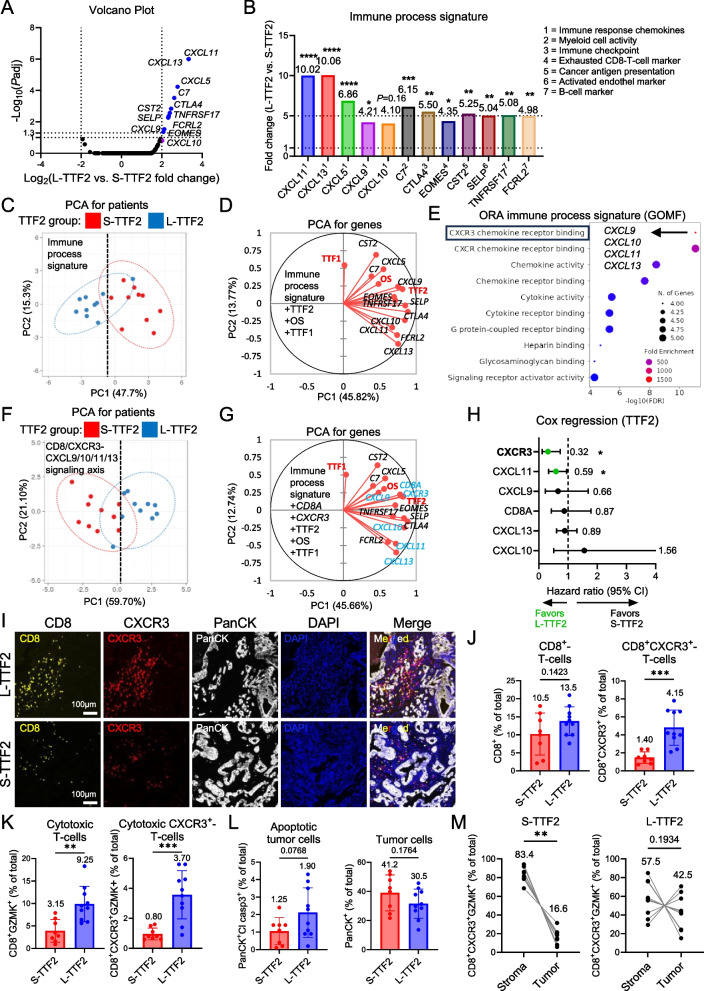
CXCR3/CD8-T-cells and CXCR3-dependent inflammatory chemokines characterize treatment-naïve PDAC tumors of patients with long-TTF2. **A** Volcano plot of L- vs. S-TTF2 differentially expressed genes (DEGs) highlighting genes with adjusted *P* < 0.05 (blue) and *CXCL10* (purple), included owing to its homology with *CXCL9/11/13*. These twelve genes represented the “immune process” signature. **B** Fold change in mean relative RNA expression of immune process genes for L- vs. S-TTF2 groups, annotated via NanoString. **C** Principal component analysis (PCA) of S-/L-TTF2 patients based on immune process genes, with 95% confidence ellipses. **D** PCA correlation circle plot linking immune process genes with patient clinical parameters (TTF1, TTF2, OS; red). **E** Overrepresentation analysis (ORA) of immune process genes highlighting the top-10 enriched GO molecular function (MF) terms (FDR < 0.05). The gene set with the highest enrichment score and corresponding genes are highlighted. **F** PCA of S-/L-TTF2 patients based on *CD8/CXCR3-CXCL9/10/11/13* inflammatory axis genes, with 95% confidence ellipses. **G** PCA correlation circle plot incorporating immune process genes, inflammatory axis genes (blue), and patient clinical parameters (TTF1, TTF2, OS; red). **H** Cox multivariate regression of inflammatory axis genes with TTF2, showing hazard ratios (HR; median indicated) and 95% confidence intervals (CI). Significant genes are marked in green. **I** Multiplexed immunofluorescence (mIF) on PDAC FFPE tissues from L-TTF2 (*n* = 10) and S-TTF2 (*n* = 8; two excluded due to sample limitations) patients for CD8, CXCR3, and PanCK, with DAPI counterstaining. Representative fluorescence images are displayed. **J-L** Quantified mIF results for (**J**) CD8⁺-T-cells vs. CD8⁺CXCR3⁺-T-cells, (**K**) cytotoxic CD8⁺GZMK⁺-T-cells vs. CXCR3⁺CD8⁺GZMK⁺-T-cells, and (**L**) PanCK⁺- vs. PanCK⁺cleaved caspase3⁺ apoptotic tumor cells (% of total cells) in S-/L-TTF2 groups. **M** Quantified mIF analysis of cytotoxic CXCR3⁺CD8⁺GZMK⁺-T-cells by tumor (PanCK^+^) vs. stromal (PanCK^−^) compartment proximity in S-/L-TTF2 groups. *P ≤ 0.05, **P ≤ 0.01, ***P ≤ 0.001, ****P ≤ 0.0001

To pinpoint specific immune drivers of this favorable outcome, we focused on the most differential genes in the treatment-naïve tumors. We identified 11 DEGs that were all significantly upregulated in L-TTF2 tumors (adjusted *P* < 0.05) and either inflammatory or immune-related (Fig. 2A-B; Fig. S3A; Table S3). Notably, several chemokines with large expression differences, such as *CXCL11 *(~ tenfold), *CXCL13* (~ tenfold), and *CXCL9* (~ fourfold) were identified. Since all belong to the same family as *CXCL10*, which was also among the highly regulated DEGs (top-16; Table S3), we further included *CXCL10* yielding a 12-gene “immune process” signature. PCA based on this signature separated L-TTF2 from S-TTF2 tumors (Fig. 2C). Expression of these genes correlated positively with each other, TTF2, and OS, but not with TTF1 (Fig. 2D), indicating coordinated immune activation in patients with better second-line outcome.

ORA of immune process genes identified top enrichment of “CXCR3 chemokine receptor binding” (Fig. 2E). KEGG pathway mapping confirmed that the key chemokines (CXCL9/10/11/13) all bind to the CXCR3 receptor (Fig. S3B) leading to CD8^+^-T-cell activation (data mining with STITCH, Fig. S3C). Correspondingly, *CXCR3* itself was upregulated in L-TTF2 tumors (Fig. S3D). External PDAC datasets supported these findings, i.e., single-cell RNA-seq showed that *CXCR3* is mainly expressed by tumor-infiltrating CD8⁺-T-cells (Fig. S3E), and TCGA data analysis found that *CXCR3* expression correlated strongly with activated/effector memory CD8⁺-T-cells (Fig. S3F, Table S4).

Based on these observations, we defined a focused six-gene inflammatory signature comprising *CXCL9/10/11/13* chemokines, *CXCR3*, and *CD8A* (Table S3). For all these genes Cohen’s d was calculated ≥ 1, supporting strong changes and sufficient statistical power (Table S3). This minimal gene combination robustly separated S-TTF2 and L-TTF2 tumors in PCA (Fig. 2F) and integrated into the immune activation process (Fig. 2G). Moreover, multivariate Cox regression indicated that high *CXCR3* (hazard ratio [HR] = 0.32, *P* = 0.011) was the most significant predictor of L-TTF2 (Fig. 2H). These data implicate a CXCR3-associated T-cell recruitment program as a key feature in treatment-naïve PDAC distinguishing patients with prolonged treatment benefit after first-line CTX.

### Cytotoxic CXCR3⁺-T-cell infiltration and tumor cell apoptosis are elevated in long-TTF2 tumors

We next validated the differences in the treatment-naïve TiME at the protein level using multiplexed immunofluorescence on the same PDAC tissues. L-TTF2 tumors showed a significantly higher frequency of CXCR3⁺CD8^+^-T-cells (~ threefold, *P* = 0.0003), but not total CD8⁺-T-cells (*P* = 0.142) (Fig. 2I-J). Likewise, cytotoxic (granzyme K⁺) CD8⁺-T-cells were more abundant in L-TTF2 tumors (~ threefold, *P* = 0.0018). This difference was even more pronounced for the CXCR3⁺ subset of cytotoxic T-cells (~ 4.6-fold, *P* = 0.0004; Fig. 2K; Fig. S4). These results confirmed an enrichment of CXCR3-expressing effector T-cells in treatment-naïve tumors of patients with L-TTF2.

L-TTF2 tumors showed a non-significant trend of increased cleaved caspase-3 staining (*P* = 0.077), but similar tumor cell counts (*P* = 0.176), indicating increased apoptosis (Fig. 2L; Fig. S4). Additionally, cytotoxic CXCR3⁺CD8⁺-T-cells were found more frequently in PanCK^+^ tumor areas from L-TTF2 than S-TTF2 patients (42.5% vs. 16.6% of these T-cells localized to tumor cells, *P* = 0.0042; Fig. 2M; Fig. S4). Thus, L-TTF2 tumors are infiltrated by more cytotoxic CXCR3⁺-T-cells that can engage with tumor cells and likely increase tumor cell apoptosis. A vigorous CXCR3⁺-T-cell response at primary diagnosis in tumor tissues therefore appears to predict also a better outcome in the advanced treatment situation.

### PBMC-based liquid biomarker profiling prior to the start of second-line chemotherapy

**Fig. 3:**
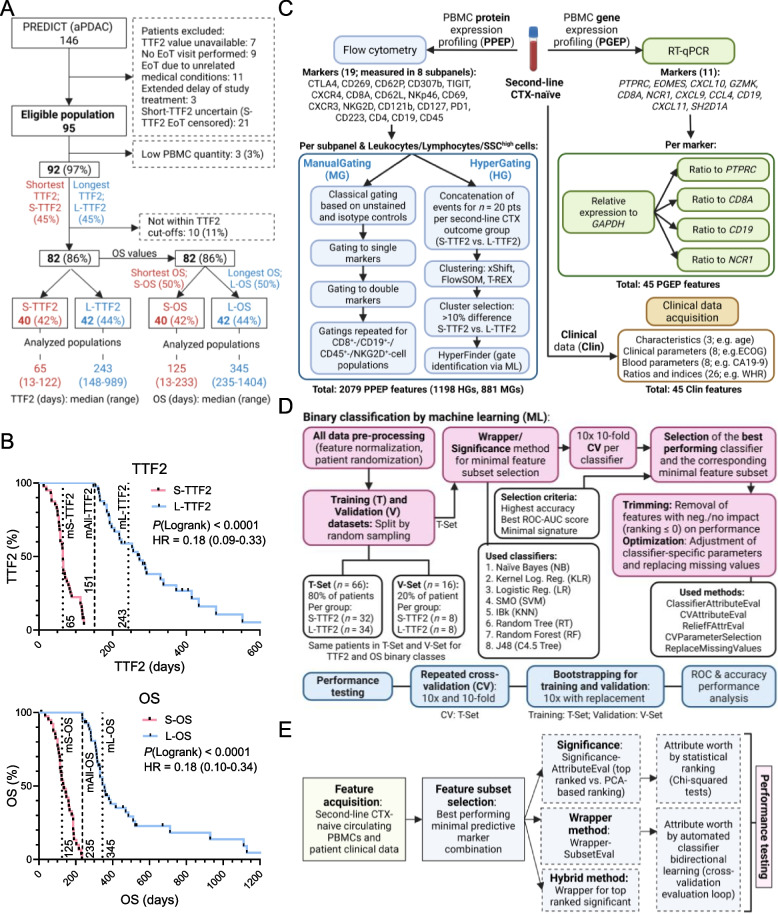
Comprehensive PBMC profiling and machine learning workflow with clinical data integration for patient outcome prediction. **A** Cohort selection for PBMC analysis. Patients with missing TTF2 values, lack of end-of-treatment (EoT) visits, unrelated EoT, delayed study treatment, or uncertain (censored) short-TTF2 (below TTF2 median of the full cohort) status were excluded. Eligible patients were stratified into S- and L-TTF2 groups (bottom/top 45^th^-percentiles; *n* = 40 and *n* = 42) and further divided by median overall survival (OS = 345 days) into S-OS (*n* = 40) and L-OS (*n* = 42) groups. **B** Kaplan–Meier curves for TTF2 and OS comparing S- and L-TTF2/OS groups. Log-rank *P*-values, hazard ratios (HR) with 95% confidence interval, and median TTF2/OS values (per cohort and for the combined group) are provided. **C** Feature selection workflow integrating clinical data (Clin) and PBMC markers from second-line treatment-naïve liquid biopsies. Flow cytometry (PPEP) and RT-qPCR (PGEP) were used to analyze PBMC markers, selected based on NanoString transcriptomic profiling. PGEP data was normalized, and ratios were calculated. PPEP data was processed via manual gating (MG) and “Hypergating” (HG), an automated clustering and machine learning (ML)-based method. CD45^+^/*PTPRC*: Leukocyte marker. CD8^+^*/CD8A:* CD8-T-cell marker. CD19^+^/*CD19*: B-cell marker. NKG2D^+^/*NCR1*: Cytotoxic cells marker. **D** ML workflow for minimal biomarker selection to predict S-/L-TTF2 (binary class) outcome. Data was split into training (80%) and validation (20%) sets (equal for TTF2 and OS). Feature selection on the training set involved statistical ranking and the Wrapper method combined with eight classifiers. The best panels were optimized and subjected to comprehensive performance testing (10× tenfold cross-validation, bootstrapping 10× with replacement). Log.: Logistic. Reg.: Regression. Neg.: Negative. **E** The ML pipeline was combined with three methods: (i) statistical ranking (top-ranked, PCA-based ranking), (ii) the Wrapper method with classifier-driven bidirectional learning, and (iii) a hybrid approach combining significant features with Wrapper selection. Schemes were generated using BioRender

We then asked whether this TiME phenotype is “mirrored” and potentially persists in circulating immune cells, providing a putative novel source of liquid predictive biomarkers. To this end, we analyzed PBMC samples collected immediately prior to the start of second-line CTX in the PREDICT trial (Fig. 1A). The PREDICT cohort was again split into short- and long-outcome groups, for both TTF2 and OS. To maximize cohort separation but also include a sensible number of patients for binary-class prediction via machine learning, TTF2 was first dichotomized at its median. Patients with uncensored TTF2 values below the median were allocated to the S-TTF2 group, whereas patients whose TTF2 exceeded the median were assigned to the L-TTF2 group, even when their TTF2 was censored (Fig. 3A). From this eligible population, top and bottom 45^th^-percentiles of TTF2 values (Fig. S5A) were subjected to the final analysis, leading to S-TTF2 (*n* = 40) and L-TTF2 (*n* = 42) cohorts (median TTF2 65 vs. 243, *P* < 0.0001; Fig. 3A-B). This resulted in a median TTF2 separation of 3.7-fold for L- vs. S-TTF2 as compared to 3.3-fold for the tissue cohort (Fig. 1C, Fig. 3B). For prediction of OS from the start of second-line treatment, the analyzed TTF2 cohort was stratified at the OS median (235 days) into S-OS (*n* = 40) and L-OS (*n* = 42) groups, respectively (median OS 125 vs. 345 days, *P* < 0.0001; Fig. 3A-B). Consistent with earlier observations, TTF2 strongly correlated with second-line CTX cycles and OS, but not TTF1 (Fig. S5B). Clinical characteristics of the “liquid collective” were largely comparable with the tissue cohorts and showed the same short vs. long group differences (Table S5). However, long group patients had also better ECOG performance status (Table S5).

Treatment-naïve tumors of patients with S-/L-TTF2 revealed substantial differences in the TiME. Thus, we asked whether a similar immune profile would exist within PBMCs prior to the start of second-line CTX using multi-parametric flow cytometry and gene expression analysis. Based on the findings from the treatment-naïve tumor tissue transcriptomics, 19 immune cell surface markers were measured by flow cytometry (PPEP: PBMC protein expression profiling) across eight panels (Table S6) and eleven immune-related genes were measured by RT-qPCR (PGEP: PBMC gene expression profiling; Fig. 3C; Fig. S5C). In total, 2079 PPEP features were extracted, combining conventional manual gating (MG; 881 features) and an automated clustering-based gating procedure (“HyperGating”, HG; 1198 features) for flow cytometry (Fig. 3C; Fig. S6A; Table S6). Importantly, each gating procedure was preceded by a sequential pre-gating strategy as indicated in the supplementary methods and Fig. S6A-C. Additionally, 45 PGEP features (normalized transcripts and calculated gene ratios) were derived from the RT-qPCR data, and 45 clinical (Clin) features (e.g., patient demographics, blood counts prior to second-line therapy; Table S7) were compiled for each patient (Fig. 3C). A unified nomenclature is shown in Fig. S6D. PBMC sample quality and yield were comparable between S- and L-TTF2 groups (Fig. S7A-C). Combined, we established a feature acquisition workflow integrating manual and automated PBMC profiling leading to a high yield of features.

### Machine learning identifies a minimal PBMC immune signature predicting TTF2 (“TTF2Pred”)

To identify the best performing minimal biomarker combination for S-/L-TTF2 (binary class) prediction, a supervised machine learning (ML) workflow was established. The data were randomly divided into training (80% of patients) and validation (20% of patients) sets (Fig. 3D). These datasets retained similar patient and sample characteristics as the full cohort (Table S8-9). We then performed feature selection on the training set by multiple strategies, including statistical filtering, a classifier-based wrapper method, and a hybrid approach (Fig. 3E; statistical filtering-based feature combinations and ranking in Table S10). Eight different classification algorithms were evaluated in repeated cross-validation (CV) to determine the optimal feature set and model (Fig. 3D).

**Fig. 4:**
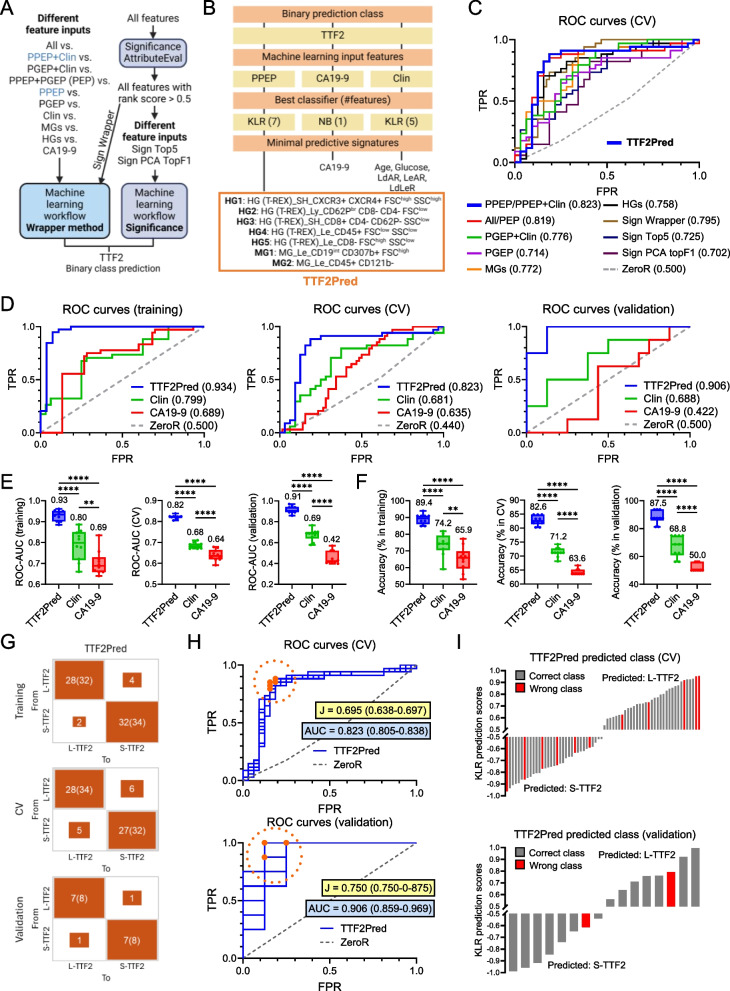
TTF2Pred: A 7-feature PBMC immune signature outperforms clinical benchmarks in predicting second-line chemotherapy outcome. **A** Comparison of the machine learning (ML) performance for S-/L-TTF2 prediction using different feature inputs from PPEP, PGEP, and Clin datasets, analyzed via three selection methods (Fig. 3E). Feature inputs leading to the best performing signature are highlighted in blue. Sign Top5: Top-5 ranked features based on statistical filtering; Sign PCA TopF1: Top-ranked features (score > 0.5) with significant squared cosine in principal component analysis (PCA) F1 axis; Sign Wrapper: Top-ranked features (score > 0.5) analyzed with the Wrapper method. **B** Optimal feature combination and corresponding ML classifier for S-/L-TTF2 prediction based on PPEP, Clin, or CA19-9. The best-performing model, termed “TTF2Pred,” was based on kernel logistic regression (KLR) and included seven PPEP features. Ratio definitions: LdAR (LDH/albumin), LeAR (leukocyte/albumin), LdLeR (LDH/leukocyte). NB: Naïve Bayes; Int: Intermediate; Br: Bright. **C** Receiver operating characteristic (ROC) curve for L-TTF2 prediction (brackets: mean ROC-AUC for S-/L-TTF2 prediction) using cross-validation (CV) based on feature inputs indicated in Fig. 4 A (except: Clin, CA19-9). **D-F** Performance metrics including (**D**) ROC curve for L-TTF2 prediction, (**E**) mean ROC-AUC, and (**F**) mean accuracy for training, CV, and independent validation. TTF2Pred was compared to the best Clin model and CA19-9. ZeroR served as a majority-class baseline predictor. Bar graph values indicate medians. **G** Confusion matrix for training, CV, and validation of TTF2Pred. Rows: Actual class; Columns: Predicted class. **H** ROC curves (L-TTF2 prediction) for repeated CV (training set) and bootstrap validation (independent cohort) based on the TTF2Pred model, with the median (range) Youden index and ROC-AUC shown. Optimal thresholds (maximized Youden index) are highlighted in orange. (**I**) Individual KLR prediction scores per patient for CV and validation. The predicted class is indicated. Schemes were generated using BioRender. **P ≤ 0.01, ****P ≤ 0.0001

We then applied the ML pipeline using different combinations of PPEP, PGEP, and Clin feature inputs lists as indicated in Fig. 4A to discover the best performing (Fig. S8A) and minimal (Fig. S8B) biomarker combination for predicting second-line CTX outcome (Table S11). This approach yielded a top-performing model consisting of a 7-feature PBMC immune signature used with a kernel logistic regression (KLR) classifier. We termed this model “TTF2Pred” (Fig. 4B; Table S12). TTF2Pred features were all derived from the PBMC protein data (PPEP) underscoring the performance and robustness of the flow cytometry-based immune profiling (Table S11-12). TTF2Pred (inputs: PPEP or PPEP + Clin) achieved the highest predictive performance metrics during CV (i.e., accuracy: 82.6%, ROC-AUC: 0.82). A similar result was obtained in an independent validation set (i.e., accuracy: 87.5%, ROC-AUC: 0.91; Fig. 4C; Fig. S8C-H).

We compared TTF2Pred with two benchmarks: Using all available clinical features (Table S7), delivering an optimal subset of five clinical features prior to the start of second-line therapy identified by the same ML pipeline, and an optimal model using CA19-9 (Fig. 4B). TTF2Pred significantly outperformed both the clinical features and CA19-9 (Fig. 4D-F; Fig. S9A-C). For example, in CV and the independent validation set, TTF2Pred achieved the highest accuracies (82.6% and 87.5%; *P* < 0.0001) compared to the clinical (71.2% and 68.8%) and CA19-9 (63.6% and 50%) ML models (Fig. 4D-F). It also had significantly higher sensitivity, specificity, and precision (Fig. S9A-C, *P* < 0.0001), with fewer false classifications (Fig. 4G; Fig. S9D). The clinical model itself performed better than CA19-9 highlighting the added value of multiple parameters (Fig. 4D-G; S9A-D). TTF2Pred model calibration was good (Fig. S9E), and TTF2Pred outperformed the ZeroR majority-class baseline classifier (Fig. S9F). TTF2Pred predictions were made with high confidence across repeated CV and validation (Fig. 4H) for most patients (Fig. 4I). These results demonstrate that a concise blood-based PBMC immune marker panel can robustly predict second-line treatment outcome in aPDAC.

### TTF2Pred model generalization and prognostic value

**Fig. 5:**
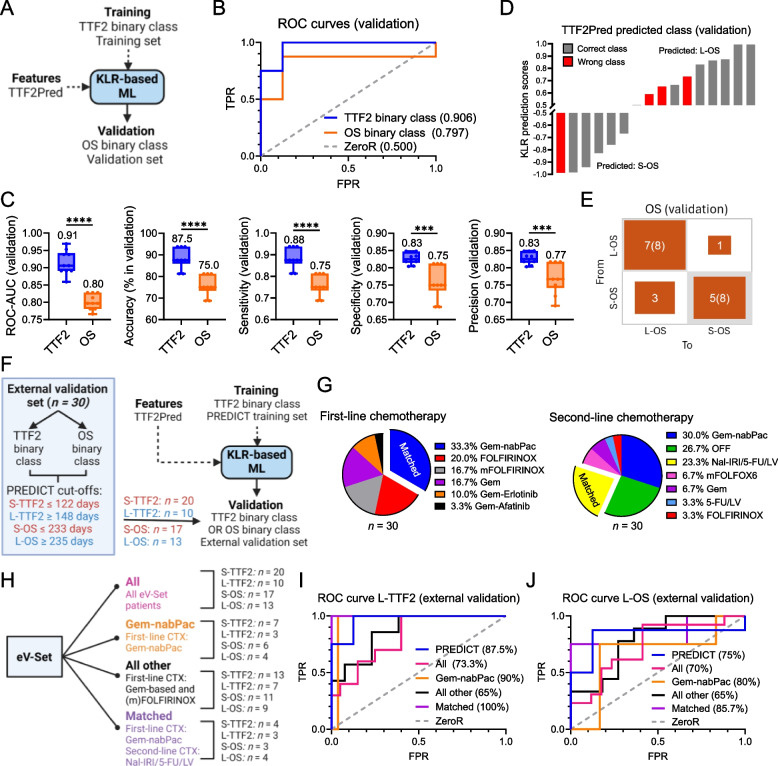
TTF2Pred demonstrates prognostic value and generalizability across diverse treatment regimens. **A** Workflow for testing TTF2Pred’s prognostic performance. The PREDICT TTF2 binary class (S-/L-TTF2) training set was used to train the model, and performance was evaluated on the independent OS binary class (S-/L-OS) validation cohort. **B-C** Performance metrics including (**B**) Receiver operating characteristic (ROC) curve for L-OS vs. L-TTF2 prediction (mean ROC-AUC for short and long prediction in brackets), (**C**) mean ROC-AUC, accuracy, sensitivity, specificity, and precision using independent validation (TTF2 vs. OS binary class prediction). ZeroR served as a majority-class baseline predictor. Median values are indicated in bar graphs. **D** Individual kernel logistic regression (KLR) prediction scores per patient for OS binary class independent validation based on TTF2Pred. Predicted classes are shown. **E** Confusion matrix for OS binary class independent validation using TTF2Pred. Rows: Actual class; Columns: Predicted class. **F** Validation workflow in an external cohort (eV-Set, *n* = 30). Patients were stratified into TTF2 and OS binary groups using the same cut-offs as in the PREDICT trial, leading to imbalanced groups (S-TTF2: *n* = 20, L-TTF2: *n* = 10, S-OS: *n* = 17, L-OS: *n* = 13). TTF2Pred was trained on PREDICT S-/L-TTF2 data and tested in the eV-Set for TTF2 and OS binary class predictions. **G** Distribution of first- and second-line treatments in the eV-Set, with PREDICT-matching regimens highlighted. **H** Stratification of eV-Set patients into four subcohorts (All, Gem-nabPac, All other, Matched) based on first-line therapy. Group sizes for OS and TTF2 binary classes are indicated. **I-J** ROC curves for (**I**) L-TTF2 and (**J**) L-OS predictions in the eV-Set cohorts from Fig. 5H based on TTF2Pred. The average accuracy for short and long groups is indicated in brackets. ZeroR was used as a baseline. Schemes were generated using BioRender. ***P ≤ 0.001, ****P ≤ 0.0001

TTF2Pred predicted S-/L-TTF2 with high performance. Our data also showed that TTF2 strongly correlates with OS, prompting us to examine whether TTF2Pred could predict OS defined from start of second-line treatment. Using our PREDICT data, we trained TTF2Pred on the TTF2 outcome and then applied it to distinguish S-OS vs. L-OS patients of the PREDICT validation cohort (Fig. 5A). The model’s performance for OS prediction was somewhat lower than for TTF2 prediction (i.e., accuracy: 75%, ROC-AUC: 0.80; *P* < 0.0001) but still achieved a high accuracy (Fig. 5B-C) with only a few false classifications (Fig. 5D-E). Thus, beyond predicting TTF2 in second-line, TTF2Pred also correlates with OS.

We further validated TTF2Pred on an independent external cohort (eV-Set), albeit limited to *n* = 30 available aPDAC patients that were in the exactly same clinical situation as the PREDICT cohort and dichotomized using the same TTF2 and OS binary class cut-offs (Fig. 5F, Fig. S10A). Patients were recruited from routine clinical practice having received various first- and second-line regimens (Fig. 5G), and blood samples were again analyzed immediately prior to start of second-line treatment. TTF2 and OS binary groups showed similar patient and sample characteristics to the PREDICT cohorts, except that CA19-9 was this time higher in the long-OS/TTF2 compared to the short-OS/TTF2 groups (Fig. S10A-D, Table S13). To evaluate the impact of different first-line treatments on TTF2Pred performance, a subgroup analysis was performed according to different first-line treatments including “All”, “Gem-nabPac”, “All other” (all except Gem-nabPac), and “Matched” (first-/second-line matched to the PREDICT therapy sequence; Fig. 5H; Table S12). TTF2Pred still had a high accuracy of 73.3% across all patients and treatments in the eV-Set (Table 1). There was a more pronounced benefit for patients who had received Gem-nabPac as first-line therapy, consistent with the context in which the model was trained (i.e., accuracy: 90%; Table 1; Fig. 5I; Fig. S10E). Notably, all patients in the “Matched” subgroup that received the same therapy sequence as in the PREDICT trial were correctly identified (Table 1). TTF2Pred could also predict OS outcome across all patients in the eV-Set (i.e., accuracy: 70%). This value was also more pronounced for the Gem-nabPac first-line cohort (i.e., accuracy: 80%; Table 1; Fig. 5J; Fig. S10F). In all cases, performance exceeded the baseline classifier ZeroR (Table 1). These findings support the predictive and prognostic utility of our PBMC-based model TTF2Pred in diverse treatment contexts and suggest a relevance of first-line Gem-nabPac therapy for the most optimal TTF2Pred performance. Clear proof of such treatment-specific prediction, however, requires regimen-specific validation in larger randomized cohorts.

**Table 1:** Comprehensive performance testing of TTF2Pred on the external validation cohorts for S-/L-TTF2 and S-/L-OS prediction

**Metric (TTF2)**	**All**	**Gem-nabPac**	**All other**	**Matched**
KLR ROC-AUC	0.820	0.857	0.868	1.000
KLR PRC-AUC	0.853	0.862	0.892	1.000
KLR Accuracy (%)	73.33	90.00	65.00	100.00
KLR Sensitivity	0.733	0.900	0.650	1.000
KLR Specificity	0.867	0.957	0.812	1.000
KLR Precision	0.852	0.925	0.825	1.000
KLR MCC	0.577	0.802	0.480	1.000
ZeroR Accuracy (%)	33.33	30.00	35.00	42.86
**Metric (OS)**	**All**	**Gem-nabPac**	**All other**	**Matched**
KLR ROC-AUC	0.738	0.667	0.788	0.833
KLR PRC-AUC	0.756	0.701	0.819	0.869
KLR Accuracy (%)	70.00	80.00	65.00	85.71
KLR Sensitivity	0.700	0.800	0.650	0.857
KLR Specificity	0.734	0.783	0.693	0.893
KLR Precision	0.737	0.800	0.715	0.893
KLR MCC	0.439	0.583	0.373	0.750
ZeroR Accuracy (%)	43.33	40.00	45.00	57.14

TTF2Pred was applied using a kernel logistic regression (KLR) classifier. ZeroR served as a majority-class baseline predictor. Reported metrics represent mean values across short and long class predictions

*MCC* Matthew’s correlation coefficient, *ROC* Receiver operating characteristic curve, *PRC* Precision-recall curve, *AUC* Area under the curve

### Circulating CXCR3^+^ inflammatory immune subsets and platelet-leukocyte aggregates define the TTF2Pred signature

**Fig. 6:**
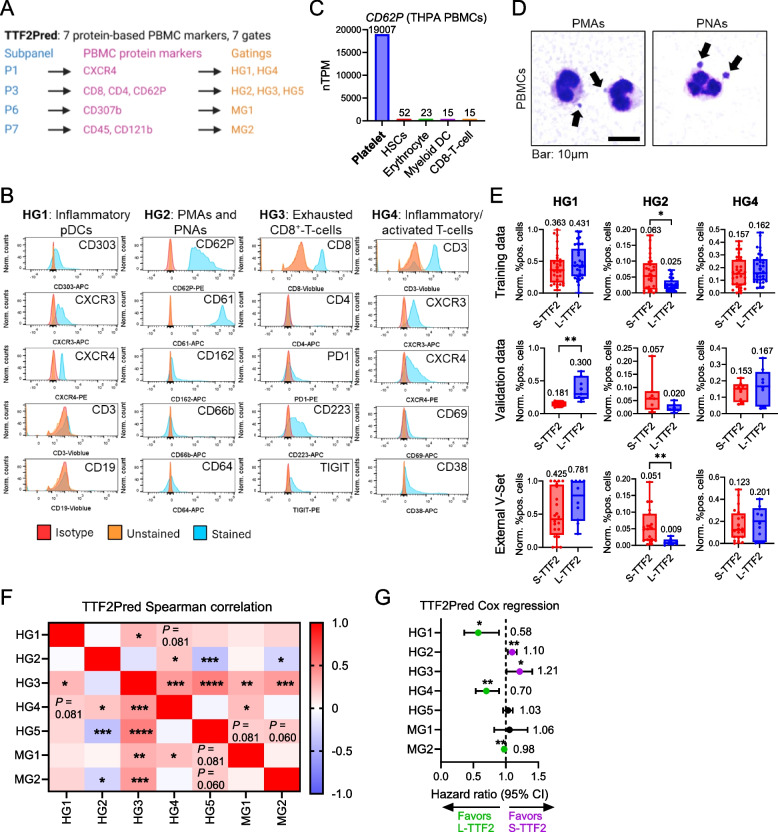
Circulating CXCR3^+^ immune cells and platelet-leukocyte aggregates define the predictive TTF2Pred immune signature. **A** Overview of the TTF2Pred marker panel, showing flow cytometry subpanels and corresponding PBMC protein markers used for final gatings. TTF2Pred includes seven PBMC markers and seven features (gatings; HG1-5, MG1-2). HG: Hypergate; MG: ManualGate. **B** Flow cytometry characterization of additional PBMC protein markers for immunophenotyping of the TTF2Pred gates HG1-4, with corresponding potential immune subsets indicated. Unstained and isotype controls were used for marker validation. Combined data (concatenated single-cell events) from *n* = 4 representative PREDICT patients. During concatenation, events were down-sampled to ensure equal representation across patients. **C** *CD62P* (P-selectin) expression in PBMC subtypes based on publicly available single-cell RNA-sequencing data from *n* = 29 samples mined with The Human Protein Atlas (THPA). The top-five expressing cell types are shown. Indicated values in the bar graph are weighted expression means over single-cells averaged across datasets. **D** Wright-Giemsa-stained PBMC sample blood smears highlighting platelet-monocyte (PMA) and platelet-neutrophil (PNA) aggregates. Arrows indicate leukocyte-bound platelets. Representative images from *n* = 4 PREDICT patients. Bar as indicated. **E** Min-max normalized frequencies (0-1) of immune subsets (features HG1/2/4) in S-/L-TTF2 groups for PREDICT training (S-TTF2: *n* = 32; L-TTF2: *n* = 34), validation (S-TTF2: *n* = 8; L-TTF2: *n* = 8), and external validation (eV-Set; S-TTF2: *n* = 20; L-TTF2: *n* = 10) cohorts. Median values are indicated. **F** Spearman correlation heatmap showing internal associations among TTF2Pred features. **G** Cox multivariate regression for individual TTF2Pred features and TTF2 association. Hazard ratios (HR) and 95% confidence intervals (CI) are shown. Indicated values are HR medians. Significant markers are highlighted in green (favorable, long-TTF2 association) or purple (unfavorable, short-TTF2 association). Schemes were generated using BioRender. *P ≤ 0.05, **P ≤ 0.01, ***P ≤ 0.001, ****P ≤ 0.0001

To gain biological insight into the TTF2Pred signature, we analyzed the immune cell subsets captured by its features (HG1-5 HyperGates, MG1-2 ManualGates) prior to the start of second-line therapy. The seven markers in the TTF2Pred panel (CXCR4, CD8, CD4, CD62P, CD307b, CD45, CD121b) defined seven flow cytometry gates (Fig. 6A) corresponding to distinct leukocyte subpopulations in the blood. The populations are characterized by protein surface markers (Fig. S11A-B) and FSC (forward scatter: cell size)/SSC (side scatter: cell granularity) parameters (Fig. S11C). Thus, to fully characterize the PBMC subpopulations, we included additional immunophenotyping markers in flow cytometry experiments on representative PREDICT PBMC samples (Fig. 6B). One key feature (HG2) was defined by high CD62P (P-selectin) expression (platelets marker, Fig. 6C) suggesting it represented platelet-bound immune cells. Blood smear analysis of PBMCs from PREDICT patients indeed revealed frequent platelet-monocyte (PMA) and platelet-neutrophil (PNA) aggregates (Fig. 6D). Our immunophenotyping confirmed that HG2 cells co-expressed CD62P^bright^/CD61^bright^ (platelet markers) with CD64^int^ monocyte and CD66b^int^ neutrophil markers, along with CD162^+^ (P-selectin ligand; Fig. 6B). This suggests an aggregate formation via engagement of CD62P with its ligand. Moreover, these cells were negative for CD4 and CD8 indicating such aggregates did not involve T-lymphocytes (Fig. S11B). HG1 and HG4 features of the PBMC profiling revealed CXCR3^+^ plasmacytoid dendritic cells (pDCs, CD303⁺) and activated CXCR3^+^-T-cells (CD3⁺CD69⁺CD38⁺) (Fig. 6B), resembling the effector CXCR3⁺-T-cells enriched in L-TTF2 treatment-naïve PDAC tissues.

We next explored the PBMC subsets related to different patient outcomes. In line with the NanoString profiling of treatment-naïve tumors, for both the PREDICT cohort and the eV-Set, L-TTF2 patients displayed higher frequencies of the CXCR3⁺ inflammatory immune cell populations (HG1, HG4; Fig. 6E). Conversely, S-TTF2 patients showed higher levels of PMAs/PNAs (HG2; Fig. 6E). Although not all differences were individually significant, features showed strongly significant inter-correlations (Fig. 6F) collectively contributing to the model’s predictive power. A Cox regression analysis confirmed that high levels of CXCR3⁺-pDCs (HG1; HR = 0.58, *P* = 0.0165) and activated CXCR3⁺-T-cells (HG4; HR = 0.70, *P* = 0.0063) were significantly associated with L-TTF2, whereas elevated PNAs/PMAs (HG2; HR = 1.10, *P* = 0.0034) and exhausted (PD1^+^CD223^+^TIGIT^+^; Fig. 6B) CD8⁺-T-cells (HG3; HR = 1.21, *P* = 0.0204) were linked to S-TTF2 (Fig. 6G). Similar, but less pronounced trends were observed for OS (Fig. S12A). The other components of the TTF2Pred panel as listed in Fig. S12B-C also aligned with biologically meaningful immune subsets but were not as strongly associated with patient outcome as individual markers (Fig. 6G).

Moreover, analysis of the three most differential features from the Significance method (all ManualGates from PPEP; Table S10; Fig. S13A) also identified CD4^+^-T-cells, CD8^+^-T-cells, and CXCR3^+^CD8^+^-T-cells with the expected fluorescence (Fig. S13B) and FSC/SSC parameter (Fig. S13C) characteristics. Of note, matching our data from treatment-naïve tumor tissues, all of these three circulating PBMC subpopulations showed higher frequencies in PBMCs of L-TTF2 patients (Table S10) prior to the start of second-line therapy.

Thus, certain therapy-naïve TiME phenotypes in combination with therapy-induced effects leave a detectable imprint in circulating PBMCs. Harnessing this tumor-PBMC immune-axis via our TTF2Pred ML-based model provides a novel approach for patient risk stratification in aPDAC using liquid biopsies.

## Discussion

Prognosis of metastatic PDAC remains poor, with median survival under one year [3]. At best, half of patients proceed to second-line therapy and many of those derive no benefit [38]. In the prospective PREDICT trial time-to-treatment failure under first-line Gem-nabPac (TTF1) did not predict second-line Nal-IRI/5-FU/LV treatment failure (TTF2) [7]. However, in line with NAPOLI-1 trial results, TTF2 and OS from the start of second-line treatment correlated with e.g., liver metastases, leukocyte levels, and CA19-9 [4]. Furthermore, in NAPOLI-1 second-line outcome varied widely upon Nal-IRI/5-FU/LV therapy (median OS 6.1 months for all patients vs. 23.4 months for long-term responders) [4], highlighting the need for biomarkers to guide individualized treatment in aPDAC. Thus, based on the PREDICT study design, we identified tissue TiME features in therapy-naïve tumors from patients with particularly long- or short-TTF2 that guided the development of a model for prediction of S-/L-TTF2 and S-/L-OS (defined from the start of second-line therapy) in liquid biopsies immediately prior to second-line therapy.

The TiME in PDAC is largely immunosuppressive [22]. Yet, treatment-naïve tumors from L-TTF2 patients exhibited an immune-enriched (“hot”) TiME with CXCR3-dependent chemokine signaling, immune cell infiltration, lymphocyte activation, and NK-cell interactions. In line with these findings, L-TTF2 tumors registered as Bailey immunogenic, whereas S-TTF2 tumors resembled a quasi-mesenchymal phenotype. This confirms and extends prior observations from Balachandran et al. in resectable PDAC that linked T-cell reactivity to PDAC survival [26]. Combined, we show that a robust tumor immune context in primary treatment-naïve tumors is crucial for second-line outcome in aPDAC.

A striking feature of L-TTF2 tumors was CXCR3⁺CD8^+^-T-cell infiltration, associated with the CXCR3 ligands CXCL9/10/11/13, and tumor cell apoptosis (non-significant trend). These CXCR3⁺-T-cells clustered more frequently in tumor cell-rich (PanCK^+^) regions, suggesting active immune-mediated tumor killing. This aligns with evidence that CXCR3-associated T-cell trafficking is crucial for anti-tumor immunity [39, 40] and that CXCR3 is required for CD8⁺-T-cell tumor homing [41]. Notably, this CXCR3-dependent “hot” TiME was evident for second- but not first-line therapy outcome. An explanation could be that first-line CTX may unmask or amplify anti-tumor immunity in a subset of tumors with per se less immunosuppressive stroma, e.g., through tumor antigen release and immunogenic cell death, to facilitate second-line therapy efficacy [42, 43]. Taken together, our data highlight a CXCR3-associated anti-tumor immune program already in the treatment-naïve TiME that appears to “prime” patients for extended responses to second-line CTX.

Our findings also suggest potential novel therapeutic approaches for a subgroup of patients with PDAC: L-TTF2 tumors exhibited cytotoxic CXCR3^+^-T-cell infiltration concomitant with increased checkpoint signaling, indicating an active immune response under regulatory inhibition rather than complete T-cell exhaustion [44, 45]. Such tumors, in contrast to PDAC in general, may be responsive to immune checkpoint inhibitors (ICIs) [46] or CXCR3 modulation via TGF-β pathway targeting [47]. Thus, a treatment-naïve “hot” tumor with cytotoxic CXCR3⁺-associated T-cell infiltration and adaptive immune resistance may represent a PDAC subset with untapped potential for immunotherapy. However, further prospective trials are required to mechanistically validate this hypothesis.

Recent PDAC research indicates a correlation between T-cell function in tissue and blood [24]. In our study, PBMCs obtained prior to second-line treatment reflected the TiME of the primary tumors. Building on tumor transcriptional profiling, we developed “TTF2Pred”, a PBMC-based, 7-feature immune signature using machine learning (ML). TTF2Pred distinguished L- vs. S-TTF2 with high predictive performance prior to second-line treatment, demonstrating that circulating immune cells carry sufficient and robust predictive information in aPDAC. TTF2Pred outperformed multivariate clinical data and CA19-9 models, supporting its clinical utility. The model's primary purpose is to identify patients who are likely to substantially benefit from Nal-IRI/5-FU/LV second-line regimen immediately prior to treatment initiation. The model further maintained high accuracy in an external validation cohort across different treatments. When stratified by the respective treatment regimens, TTF2Pred achieved optimal performance (100% accuracy) in the"matched"subgroup receiving the identical PREDICT trial regimens (first-line Gem-nabPac, second-line Nal-IRI/5-FU/LV), and strong performance (90% accuracy) in patients receiving first-line Gem-nabPac regardless of second-line therapy. This pattern suggests that the model captures treatment-specific biological responses rather than purely prognostic features. The superior performance of TTF2Pred in Gem-nabPac-treated patients may reflect either training set characteristics or genuine biological effects of this regimen on immune priming. Indeed, paclitaxel was shown to induce a cytotoxic anti-tumor immune response by reducing the secretion of immunosuppressive cytokines [25], and gemcitabine boosted antigen presentation leading to ICD induction [48]. In addition, TTF2Pred also effectively stratified patients by OS, suggesting that the captured immune features have prognostic value and reflect disease aggressiveness, albeit with lower performance than its primary predictive function. However, a clear limitation is the single-arm design of PREDICT, which inherently limits our ability to definitively distinguish predictive from prognostic effects for the second-line Nal-IRI/5-FU/LV therapy. This has to be evaluated in larger, randomized prospective trials. If validated prospectively, the TTF2Pred model could guide triaging, by identifying patients who may benefit from early palliative care rather than chemotherapy. Since blood immune profiling is minimally invasive and feasible to repeat, enabling monitoring during treatment cycles, further advantages may be harnessed in the future.

The circulating PBMC immune features underlying TTF2Pred indicated whether patients exhibited an anti-tumor immune response or a pro-inflammatory state. In L-TTF2 patients, circulating CXCR3⁺CD8^+^-T-cells were still elevated after failure of first-line CTX, mirroring the immune-enriched nature of the treatment-naïve tumor tissues with e.g., cytotoxic CXCR3^+^CD8^+^-T-cell infiltration. Circulating CD8^+^-T-cells, CD4^+^-T-cells, and CXCR3^+^CD8^+^-T-cells also showed the strongest difference between S- vs. L-TTF2 in the statistical-filtering approach. Indeed, recent studies also suggest that PBMCs reflect cancer immune cell dynamics [49, 50]. S-TTF2 patients also showed high levels of CD62P⁺ (P-selectin) monocytes and neutrophils, indicative of inflammatory platelet-leukocyte aggregates (PMAs/PNAs), which are known to facilitate tumor progression, e.g., by aiding metastatic spread [51, 52]. Thus, a concerted analysis of therapy-naïve tumors to guide liquid biopsy biomarker selection via ML is a powerful tool to triage patients according to therapy outcome and prognosis, even in aPDAC.

We must also acknowledge further limitations concerning our study design: We have deliberately selected patients from the extremes of the outcome distribution and thus successfully identified strong biological signals but also captured expected differences in clinical prognostic factors between groups. Patients with poor outcomes naturally presented with more liver metastases, worse ECOG status, and higher CA19-9 levels. Nevertheless, our comparative machine learning approach, the external validation results, and the biological coherence between tissue and blood suggested that the identified TTF2Pred PBMC immune signature provides predictive information beyond these clinical variables. In line, the multivariate Cox regression analyses for the signature’s individual immune markers were not adjusted for the clinical factors, such as liver metastases and CA-19-9. The observed associations may therefore partially reflect imbalances in disease severity factors rather than immune mechanisms alone. Thus, future prospective validation in unselected patient populations with comprehensive multivariable modeling may help to definitively establish the independent predictive value of these immune signatures and their applicability across the full spectrum of disease presentations. We also see the limitation in the cohort sizes used to establish and validate the TTF2Pred model, in particular for the validation sets. Although model performance was successfully verified in independent validation cohorts, larger studies are needed to confirm TTF2Pred generalizability. Furthermore, TTF2Pred was developed as a translational project of the PREDICT trial for Nal-IRI/5-FU/LV. Its performance in other CTX settings was tested only in very small subcohorts of the external validation set and does require further assessment with much larger cohort sizes. We also wish to indicate that the S- and L-TTF2 cohorts from liquid and tissue analyses are not fully identical, since liquid cohorts were separated to maximize patient numbers and cohort differences for robust training of predictive machine learning models. For the same reason, S- and L-TTF2 primary tissues were obtained from patients (*n* = 10 per group) at initially different disease stages, indicated by a higher incidence of liver metastases in S-TTF2 across cohorts. We further acknowledge that the size of the tissue analysis cohorts may be a limitation and moderate changes in gene expression may have been missed. Yet, our focus was on strong TiME alterations with Cohen’s d ≥ 1 to identify putative biomarkers. We recognize that spatial heterogeneity during tissue sampling by laser capture microdissection may have further introduced some bias in the NanoString analysis. To offset such errors as much as possible, sampling sites were randomly chosen, and tumor as well as stroma content (percentage) was stringently adjusted during microdissection to generate comparable sampling results (Table S2). Moreover, PBMC immune dynamics at diagnosis could not be assessed in the prospective PREDICT cohort due to the start of the study at progression after first-line treatment and unavailability of these samples at that timepoint. Thus, the recurrent immune features detected in PBMCs prior to the start of second-line therapy may reflect both therapy-naive TiME imprinting and changes driven by first-line chemotherapy. To discern these effects, further studies in PBMCs prior to first-line therapy are needed. Nevertheless, our model appears robust, since we could confirm our findings in a completely independent external validation set, even with distinct chemotherapy regimens. In addition, TTF2 showed a positive correlation with OS, despite OS encompassing both treatment-line and treatment-free intervals. Since PREDICT was a single-arm trial with the primary aim to define whether TTF1 would predict TTF2 in aPDAC, we cannot finally determine whether our model is more predictive for second-line Nal-IRI/5-FU/LV or a prognostic tool. This has to be evaluated in larger prospective trials.

While current clinical strategies emphasize the importance of the first-line treatment regimen (“hit hard and early”), our findings highlight a subpopulation that clearly seems to benefit from second-line therapy. This group can be identified using the TTF2Pred model. However, the specific contribution of each treatment-line and regimen requires further investigation. Yet, our analytical pipeline is potentially adaptable to other clinical trials and diverse tumor types, integrating multimodal data to uncover novel liquid biomarkers for triaging patients into treatment cohorts.

## Conclusions

An immune-responsive TiME in treatment-naïve PDAC that is integrated with therapy-induced changes leaves an “imprint” in PBMCs that outlives first-line chemotherapy and can be harnessed to predict S-/L-TTF2 as well as S-/L-OS in aPDAC. Patients with CXCR3⁺-T-cell enriched (immunologically active) treatment-naïve tumors are associated with significantly longer TTF2. They were also identifiable by a concise ML-based immune signature in their blood immediately prior to second-line treatment. Our comprehensive ML pipeline and the findings of this study may allow for prognostic triaging of eligible patients into a second-line Nal-IRI/5-FU/LV therapy cohort with clear favorable outcome, as well as indicate patient subsets that could hypothetically profit from targeted therapeutic avenues, such as ICI co-treatment.

## Supplementary Information

Additional file 1: Supplementary Tables S1–S13 supporting the data presented in the main text

Additional file 2: Supplementary Figures S1–S13 supporting the data presented in the main text

Additional file 3. Supplementary methods supporting the data presented in the main text

## Data Availability

Generated data are provided within the manuscript, in supplementary information files, or available upon reasonable request from the authors. Publicly accessible datasets are referenced and detailed in the main or supplementary methods.

## Abbreviations

5-FU: 5-Fluorouracil
aPDAC: Advanced Pancreatic Ductal Adenocarcinoma
AUC: Area Under the Curve
CA19-9: Carbohydrate Antigen 19-9
CTX: Chemotherapy
CV: Cross‑Validation
DEGs: Differentially Expressed Genes
ECOG: Eastern Co‑operative Oncology Group
FDR: False Discovery Rate
FFPE: Formalin‑Fixed Paraffin‑Embedded
FSC: Forward scatter
Gem-nabPac: Gemcitabine Plus Nanoliposomal Paclitaxel
GO: Gene Ontology
GSEA: Gene Set Enrichment Analysis
HG: HyperGating
HR: Hazard Ratio
ICI: Immune Checkpoint Inhibitor
KLR: Kernel Logistic Regression
LV: Leucovorin
MG: ManualGating
mIF: Multiplexed Immunofluorescence
ML: Machine Learning
Nal-IRI: Nanoliposomal Irinotecan
NES: Normalized Enrichment Score
NK: Natural Killer
ORA: Overrepresentation Analysis
OS: Overall Survival
PBMC: Peripheral Blood Mononuclear Cell
PCA: Principal Component Analysis
PDAC: Pancreatic Ductal Adenocarcinoma
pDC: Plasmacytoid Dendritic Cell
PGEP: PBMC Gene Expression Profiling
PMA: Platelet-Monocyte Aggregate
PNA: Platelet-Neutrophil Aggregate
PPEP: PBMC Protein Expression Profiling
ROC: Receiver Operating Characteristic
SSC: Side scatter
TCGA: The Cancer Genome Atlas
T (i) ME: Tumor (immune) Microenvironment
TTF1: Time-to-Treatment Failure of First-Line Therapy
TTF2: Time-to-Treatment Failure of Second-Line Therapy
