# Supplementary material for: An immune responsive tumor microenvironment imprints into PBMCs and predicts outcome in advanced pancreatic cancer: lessons from the PREDICT trial
Source: Mol Cancer. 2025 Jul 22;24:202. doi: 10.1186/s12943-025-02406-7 (PMC12281745; doi:10.1186/s12943-025-02406-7)
Supplement: Supplementary file 2 — Additional file 2: Supplementary Figures S1–S13 supporting the data presented in the main text [file 12943_2025_2406_MOESM2_ESM.pdf]

Fig. S1

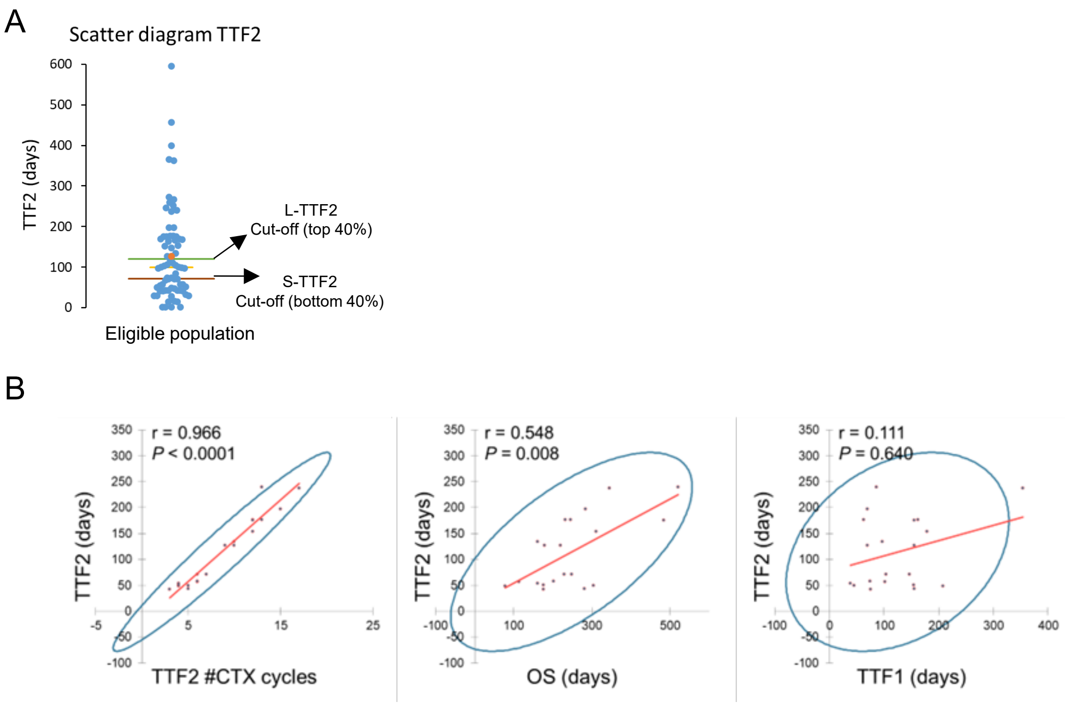

Fig. S1: Characterization of the PREDICT tissue analysis cohort. (**A**) Distribution of TTF2 values in eligible patients (*n=77)* from the PREDICT tissue cohort, highlighting the upper (green line) and lower (red line) 40th percentiles used as cut-offs. The median TTF2 is shown in orange. (**B**) Spearman correlation analysis of TTF2 with the number of second-line chemotherapy (CTX) cycles administered, overall survival (OS) from study baseline, and time to treatment failure of the first-line treatment (TTF1) for the PREDICT tissue cohort (*n*=20*)*.

Fig. S2

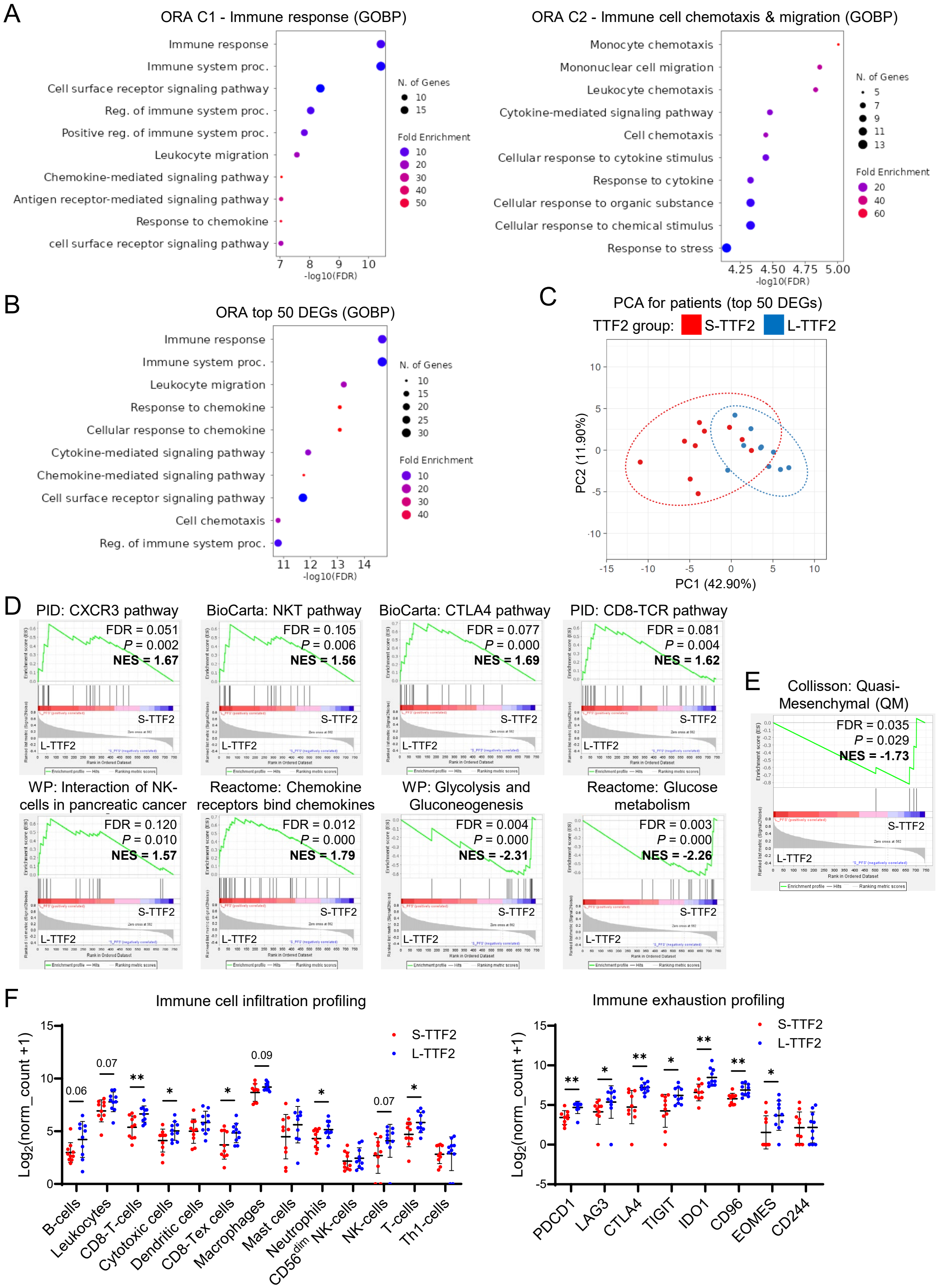

Fig. S2: Exploratory transcriptomic analysis reveals an immune-enriched tumor microenvironment in treatment-naïve PDAC tumors of long-TTF2 patients. (**A-B**) Overrepresentation analysis (ORA) (**A**) of clusters C1/C2 from hierarchical clustering (Fig. 1D) with derived cluster annotation and (**B**) of the top 50 S- vs. L-TTF2 differentially expressed genes (DEGs) across GO Biological Process (BP) gene sets. The top 10 enriched pathways are shown, respectively. Reg.: Regulation; Proc.: Process. (**C**) Principal component analysis (PCA) of top 50 S- vs. L-TTF2 DEGs with 95% confidence ellipses. (**D**) Selected gene set enrichment analysis (GSEA) plots for MSigDB cellular pathway gene sets (datasets: WikiPathways, WP; Gene Ontology, GO; Pathway Interaction Database, PID; BioCarta; Reactome). Similarity/q-value cut-offs: 0.5/0.2 (P<0.05). TCR: T-cell receptor. (**E**) GSEA of PdacR PDAC subclass signatures enriched in S-TTF2 group tumors (FDR<0.05). (**F**) Relative RNA expression of NanoString immune cell gene signatures and immune checkpoint/exhaustion markers in S- and L-TTF2 tumors. Tex: Exhausted T-cells. Dim: Weak fluorescence intensity. PREDICT tissue analysis cohort: n=20. *P≤0.05, **P≤0.01.

Fig. S3

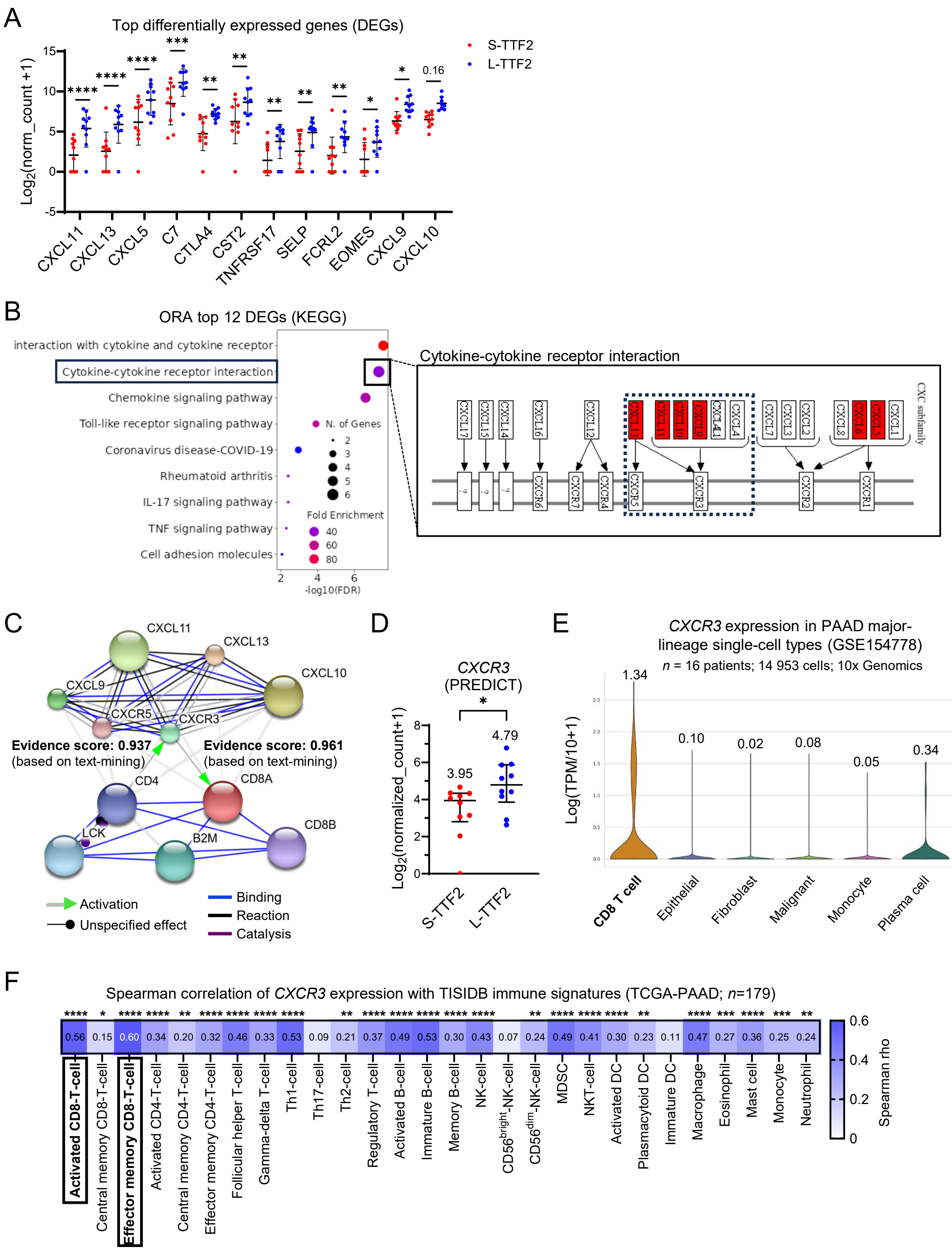
A
Top differentially expressed genes (DEGs)
S-TTF2
L-TTF2
Log2(norm_count +1)
CXCL11
CXCL13
CXCL5
C7
CTLA4
CST2
TNFRSF17
SELP
FCRL2
EOMES
CXCL9
CXCL10
0.16
B
ORA top 12 DEGs (KEGG)
interaction with cytokine and cytokine receptor
Cytokine-cytokine receptor interaction
Chemokine signaling pathway
Toll-like receptor signaling pathway
Coronavirus disease-COVID-19
Rheumatoid arthritis
IL-17 signaling pathway
TNF signaling pathway
Cell adhesion molecules
N. of Genes
Fold Enrichment
-log10(FDR)
Cytokine-cytokine receptor interaction
CXC subfamily
C
CXCL11
CXCL13
CXCL10
CXCL9
CXCR5
CXCR3
Evidence score: 0.937
(based on text-mining)
Evidence score: 0.961
(based on text-mining)
CD4
CD8A
CD8B
LCK
B2M
Activation
Unspecified effect
Binding
Reaction
Catalysis
D
CXCR3
(PREDICT)
3.95
4.79
Log2(normalized_count+1)
S-TTF2
L-TTF2
E
CXCR3 expression in PAAD major-lineage single-cell types (GSE154778)
n = 16 patients; 14 953 cells; 10x Genomics
Log(TPM/10+1)
1.34
0.10
0.02
0.08
0.05
0.34
CD8 T cell
Epithelial
Fibroblast
Malignant
Monocyte
Plasma cell
F
Spearman correlation of CXCR3 expression with TISIDB immune signatures (TCGA-PAAD; n=179)
0.56 0.15 0.60 0.34 0.20 0.32 0.46 0.33 0.53 0.09 0.21 0.37 0.49 0.53 0.30 0.43 0.07 0.24 0.49 0.41 0.30 0.23 0.11 0.47 0.27 0.36 0.25 0.24
Activated CD8-T-cell
Central memory CD8-T-cell
Effector memory CD8-T-cell
Activated CD4-T-cell
Central memory CD4-T-cell
Effector memory CD4-T-cell
Follicular helper T-cell
Gamma-delta T-cell
Th1-cell
Th17-cell
Th2-cell
Regulatory T-cell
Activated B-cell
Immature B-cell
Memory B-cell
NK-cell
CD56bright-NK-cell
CD56dim-NK-cell
MDSC
NKT-cell
Activated DC
Plasmacytoid DC
Immature DC
Macrophage
Eosinophil
Mast cell
Monocyte
Neutrophil
Spearman rho

Fig. S3

Fig. S3: Enrichment of the CXCR3/CD8-CXCL9/10/11/13 pathway in treatment-naïve PDAC tumors of long-TTF2 patients. (**A**) Relative mRNA expression (NanoString) of the top eleven differentially expressed genes (DEGs) between tumors of S- and L-TTF2 patients (adjusted *P*<0.05). *CXCL10* was included owing to its homology with *CXCL9/11/13* and its ranking among the top-16 DEGs. These twelve genes represented the “immune process” signature. (**B**) Overrepresentation analysis (ORA) of the immune process genes, showing the top 10 enriched KEGG pathways. The cytokine-cytokine receptor interaction pathway, with the second-lowest false discovery rate (FDR), is highlighted. (**C**) Protein interaction network for the CD8/CXCR3-CXCL9/10/11/13 inflammatory-signaling axis with most relevant associated proteins using the STITCH database. Evidence scores (text-mining) are shown for functional links, indicating activation. (**D**) Relative RNA expression (NanoString) of *CXCR3* in S- and L-TTF2 tumors (medians shown). (**E**) *CXCR3* expression across pancreatic adenocarcinoma (PAAD) major-lineage single-cell types using publicly available single-cell RNA-sequencing data (GSE154778 dataset, n=16). CD8-T-cells, showing the highest *CXCR3* expression, are highlighted. Mean expression across single-cells is indicated. (**F**) Spearman correlation of *CXCR3* expression with TISIDB immune cell signatures based on publicly available TCGA-PAAD data (n=179). Spearman coefficients (rho) are shown. The two immune cell subtypes with the highest rho values are highlighted. Bright: Bright fluorescence intensity; Dim: Low fluorescence intensity. PREDICT tissue analysis cohort: *n*=20. *P≤0.05, ***P*≤0.01, ****P*≤0.001, *****P*≤0.0001.

Fig. S4

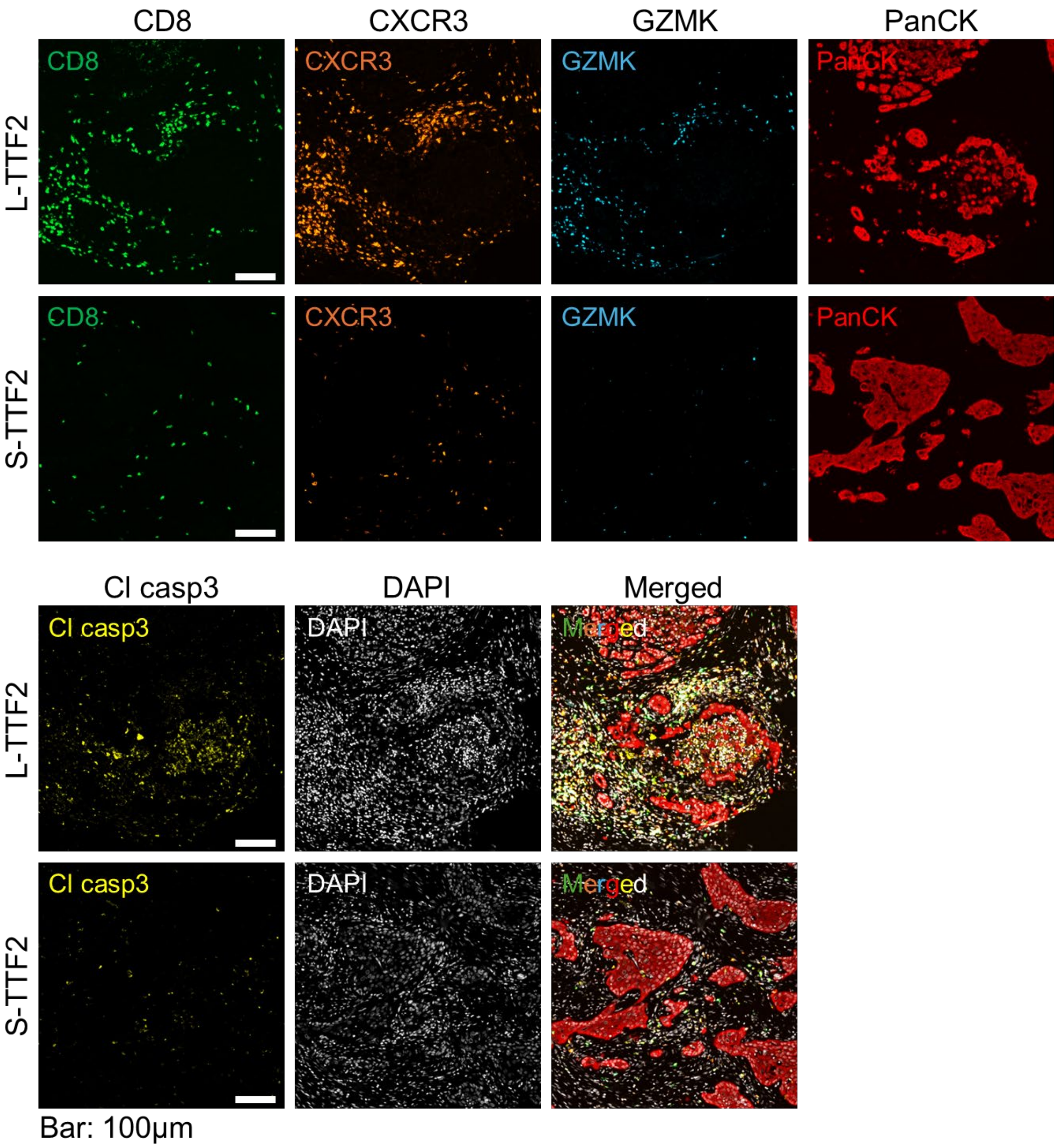

Fig. S4: PDAC tumors of long-TTF2 patients have increased infiltration of cytotoxic CXCR3$^+$CD8$^+$-T-cells. Multiplexed immunofluorescence (mIF) analysis of PDAC FFPE tissues from L- (n=10) and S-TTF2 (n=8) patients. Two S-TTF2 specimens were excluded due to tissue limitations. Staining was performed for CD8, CXCR3, GZMK (granzyme K), PanCK (pan-cytokeratin), and cleaved caspase-3 (Cl casp3; apoptotic cells) protein markers, with DAPI counterstaining. Representative single and color-merged fluorescence images are shown. Scale bar as indicated.

Fig. S5

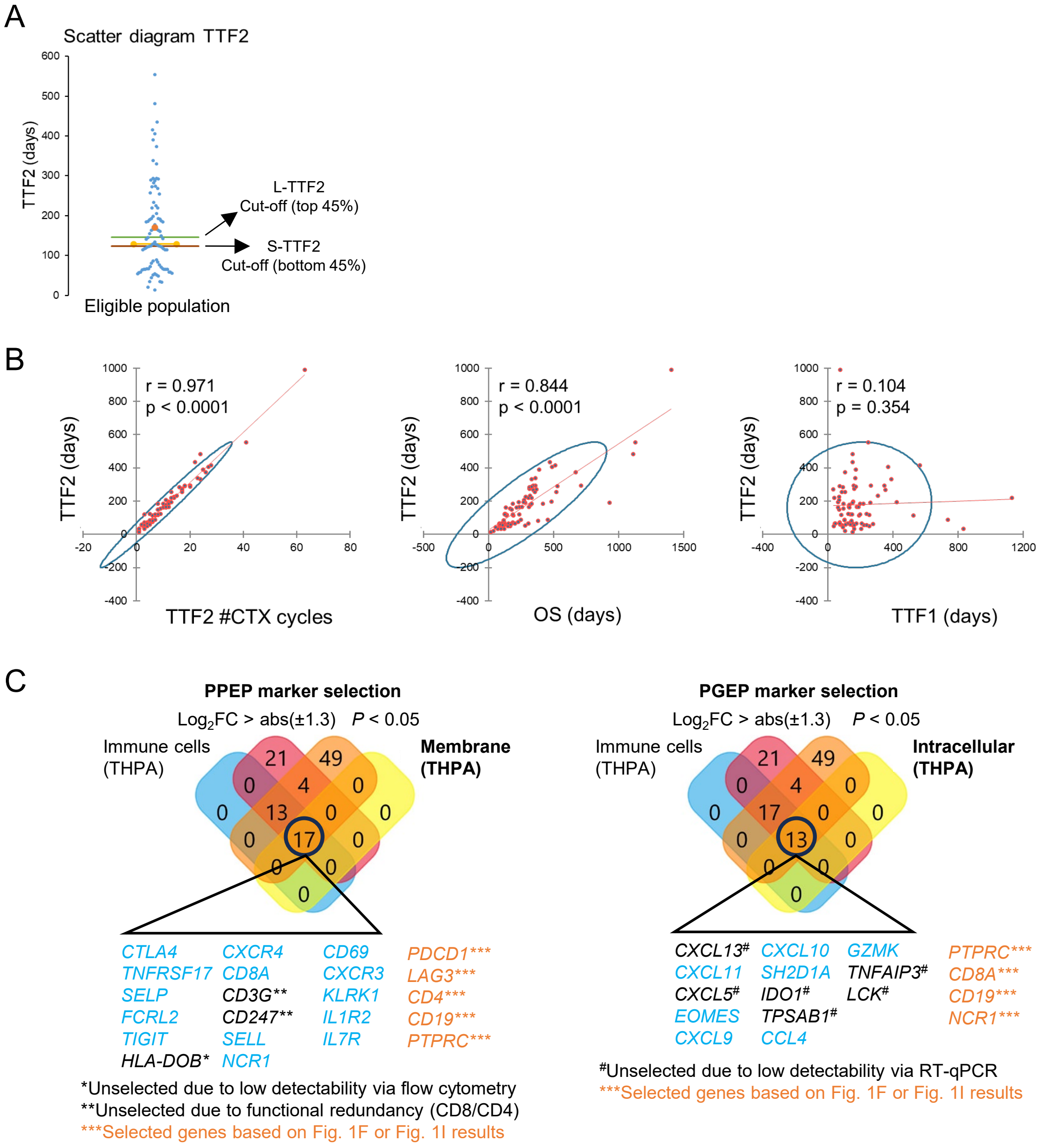

Fig. S5: Characterization of the PREDICT PBMC analysis cohort and molecular markers for PBMC profiling. (**A**) Distribution of TTF2 values in eligible PREDICT PBMC cohort patients *(n*=95). The top (green) and bottom (red) 45th-percentile cut-offs define S- and L-TTF2 groups. The median TTF2 (orange) is indicated. (**B**) Spearman correlation of TTF2 with second-line chemotherapy (CTX) cycles, overall survival (OS) from study baseline, and first-line treatment failure (TTF1) in the analyzed PBMC cohort *(n*=20). (**C**) Selection of PBMC gene expression profiling (PGEP) and PBMC protein expression profiling (PPEP) markers based on NanoString transcriptomic data from the PREDICT tissue cohort *(n*=20). Overlapping genes were identified using immune cell markers according to annotations from The Human Protein Atlas (THPA), S- vs. L-TTF2 differential expression thresholds (log_2fold change(FC)>1.3 or <−1.3; *P*<0.05), and intracellular (only for PGEP) or membrane (only for PPEP) protein localization as defined by THPA. Non-selected overlapping genes are annotated and reasons indicated. Final markers for comprehensive PBMC profiling were selected based on Venn overlap (blue) or transcriptional profiling results from Fig. 1F or Fig. 1I (orange).

Fig. S6

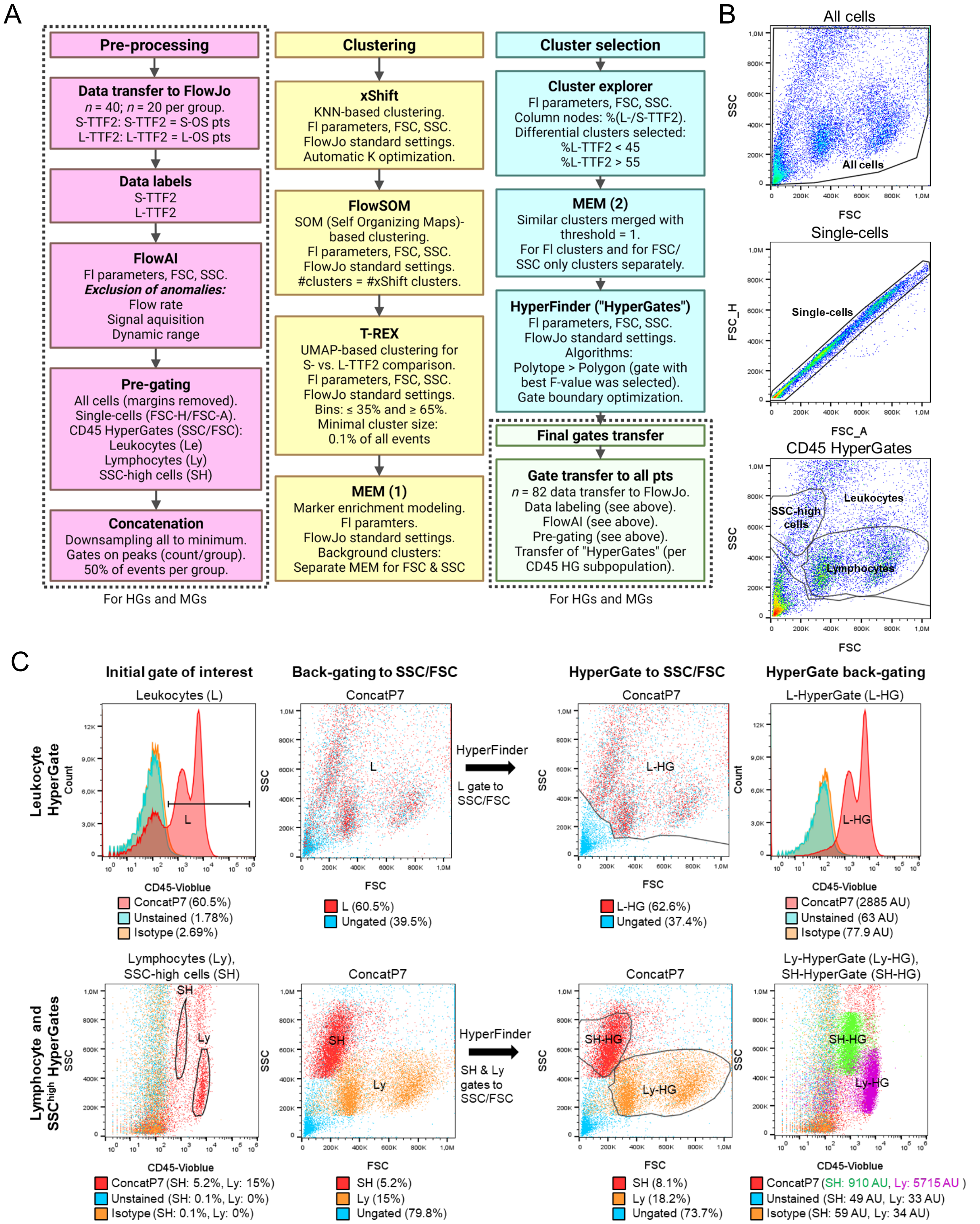

D

Nomenclatures:
PPEP: Gating procedure (clustering algorithm)_CD45 HyperGate_MEM enriched markers (HG) OR manual markers (MG)
PGEP: Ratio (R) OR relative expression (Rel)_*marker1*_*marker2* (second marker only for ratios)
Clin: All feature names are defined in Table S7

Fig. S6

Fig. S6: Feature acquisition pipeline for PBMC protein expression profiling and feature nomenclature. (**A**) PBMCs of the PREDICT cohort were analyzed by flow cytometry using both ManualGating (MG) and automated HyperGating (HG) approaches. The workflow included pre-processing steps and the application of MG and HG methods to 20 patients with the shortest (S-) and longest (L-) TTF2 (only patients with matched S-/L-TTF2 and S-/L-OS), respectively. The final gates from both procedures (HGs, MGs) were then applied to the full PREDICT PBMC cohort (*n*=82) across eight flow cytometry subpanels. MG relied on predefined gating strategies using unstained and isotype controls. The HG procedure automated clustering of single-cell events using multiple algorithms followed by the selection of most differential clusters between S- and L-TTF2 groups. Final HGs were generated from selected clusters by machine learning (ML)-based back-gating with HyperFinder in FlowJo. UMAP: Uniform manifold approximation and projection; Fl: Fluorescence; FSC: Forward scatter; SSC: Side scatter; KNN: K-nearest neighbors; T-REX: Tracking responders expanding; MG: ManualGate; HG: HyperGate; Pts: Patients. (**B**) General pre-gates from data pre-processing that were applied to all HGs and MGs (all cells → single cells → CD45 HyperGates: leukocytes, lymphocytes, SSC-high cells). Data represent concatenated events across 82 patients in the PREDICT PBMC cohort. (**C**) Transfer of leukocyte (L), lymphocyte (Ly), and SSC-high (SH) populations from CD45-based fluorescence histograms to SSC/FSC parameters. First, populations of interest were identified using CD45-Vioblue fluorescence histograms in the concatenated flow cytometry subpanel P7 (ConcatP7; includes the CD45 marker) and validated with unstained and isotype controls. Identified populations were then back-gated to SSC/FSC parameters (left panel) for comparison with the final HGs (right panel). HyperGating was applied to transfer the gates to SSC/FSC parameters, allowing identification of distinct immune subsets (L-HG, Ly-HG, SH-HG) without CD45 staining. The process was validated by back-gating HGs to CD45 fluorescence histograms in ConcatP7 (right panel). Frequencies of gated populations are indicated in brackets. Fluorescence intensities for back-gated HGs are shown in arbitrary units (AU). Data represent concatenated events across 82 patients (PREDICT PBMC cohort). (**D**) Nomenclature for PPEP, PGEP, and Clin features. PPEP markers were determined using automated Marker Enrichment Modeling (MEM) scores for HGs and manually selected gate parameters for MGs. PGEP features have one marker for relative expression-based (Rel) features and two markers for ratio-based (R) features. Clin features were assigned individualized names as listed in Table S7.

Fig. S7

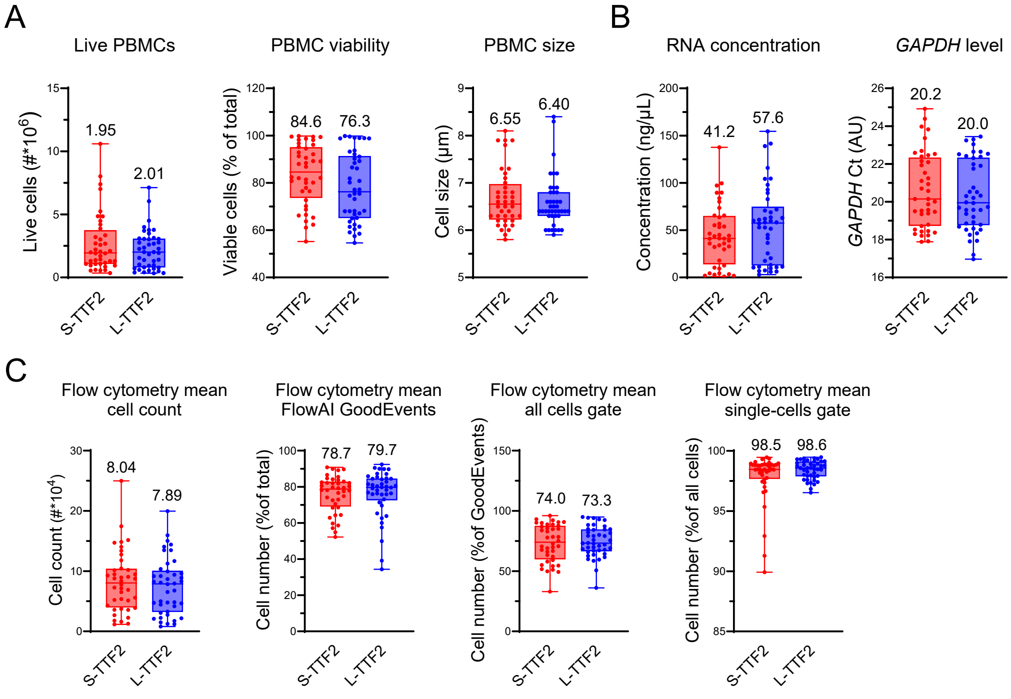

Fig. S7: PBMC profiling: quantity and quality assessment. (**A-C**) Quantification and quality control of PBMC samples from S- and L-TTF2 patients in the PREDICT PBMC analysis cohort (*n*=82). Median values are indicated. (**A**) Overall PBMC yield and viability, including total live PBMC count, viability percentage, and average PBMC size. (**B**) Quality and quantity assessment of PBMC gene expression profiling (PGEP), including RNA yield from PBMCs and mean *GAPDH* cycle threshold (Ct) values across measured plates from RT-qPCR. (**C**) Protein expression profiling (PPEP) quantity assessment, including post-flow cytometry cell count, percentage of retained cells after automated cleaning with FlowAI, and percentage of cells in key pre-gating steps (all cells, single-cells), shown relative to the respective parental gate. All PPEP data represent means across flow cytometry panels.

Fig. S8

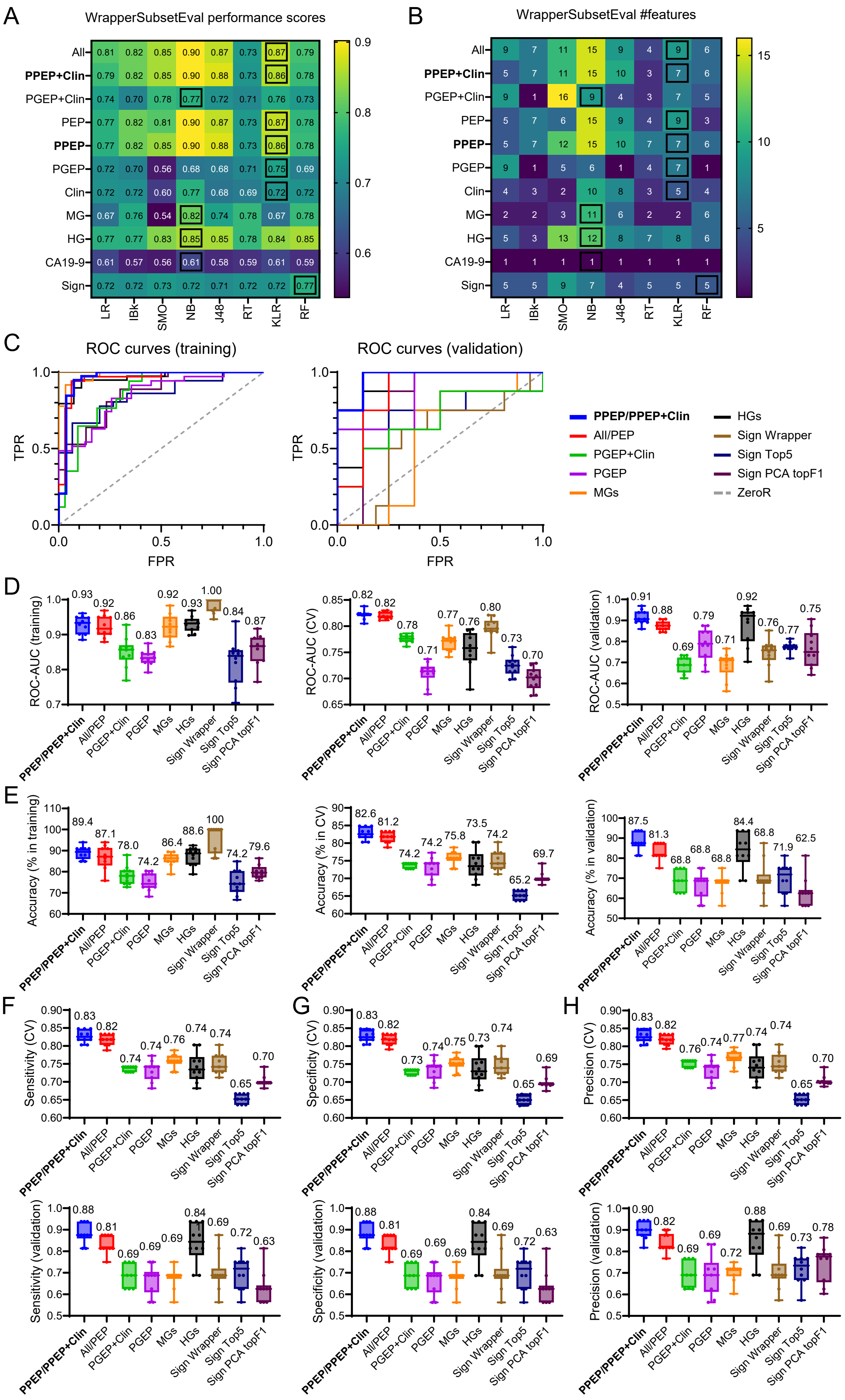
A
WrapperSubsetEval performance scores
B
WrapperSubsetEval #features
C
ROC curves (training)
ROC curves (validation)
PPEP/PPEP+Clin
All/PEP
PGEP+Clin
PGEP
MGs
HGs
Sign Wrapper
Sign Top5
Sign PCA topF1
ZeroR
D
E
F
G
H

Fig. S8

Fig. S8: PPEP-based biomarker combinations identified with the Wrapper method show the best performance for predicting second-line chemotherapy outcome in aPDAC. (**A-B**) WrapperSubsetEval algorithm performance scores (**A**) and feature subset sizes (**B**) for minimal biomarker combinations predicting S- and L-TTF2 binary classes, evaluated using different feature-input lists (Fig. 4A) and machine learning (ML) classifiers (Fig. 3D). The best-performing and thus further validated minimal predictive feature combinations with corresponding ML algorithms (optimal models) are highlighted (black boxes). (**C**) Receiver operating characteristic (ROC) curves for predicting L-TTF2 (positive class) using the optimal ML models for each feature selection method and feature input list (Fig. 4A). ZeroR, which predicts S- and L-TTF2 based on the majority class, served as the baseline, with constant ROC curves across compared models. (**D-H**) Mean performance metrics for S-/L-TTF2 binary classification based on the optimal ML models for each feature selection method and feature input list (Fig. 4A), including (**D**) ROC-AUC, (**E**) accuracy, (**F**) sensitivity, (**G**) specificity, and (**H**) precision for training (only ROC-AUC, accuracy), cross-validation (CV), and independent validation. Median values from repetitions (CV) and bootstrapping (training, validation) are indicated in bar graphs. The best-performing feature inputs for the Wrapper method (PPEP and PPEP+Clin) are highlighted in bold. The number of patients per S-/L-TTF2 group in training and validation sets are indicated in Fig. 3D.

Fig. S9

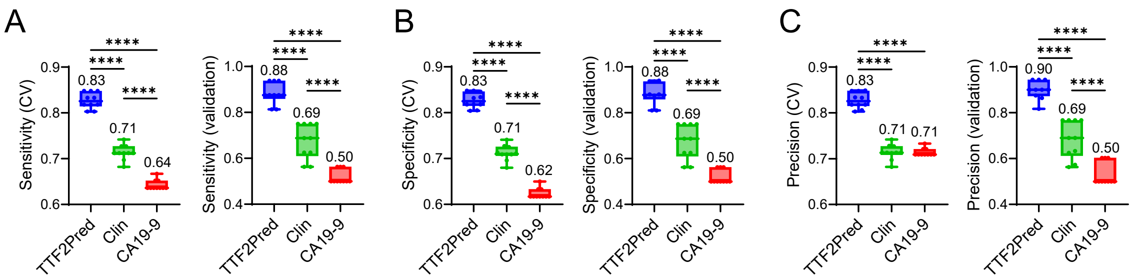

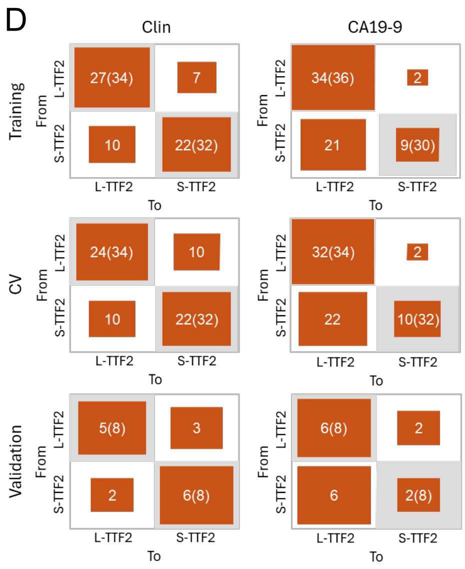

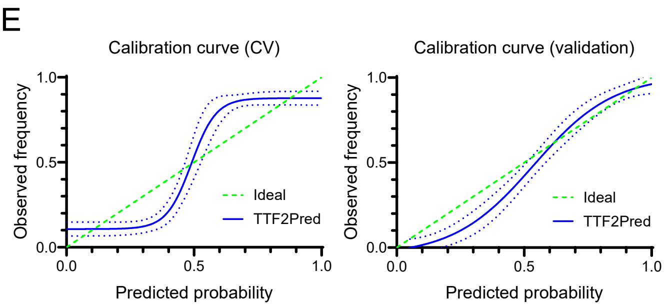

F

ZeroR performance scores

All feature inputs (constant)	Accuracy (%)	ROC-AUC
Training (TTF2)	51.52	0.50
CV (TTF2)	51.52	0.44
Validation (TTF2 & OS)	50.00	0.50

Fig. S9: The TTF2Pred model outperforms clinical benchmarks in predicting second-line chemotherapy outcome. (**A-C**) Mean performance metrics for S-/L-TTF2 binary class prediction, including (**A**) sensitivity, (**B**) specificity, and (**C**) precision, following cross-validation (CV) and independent validation. TTF2Pred was compared to the best multiparameter clinical (Clin) and CA19-9 model, respectively. The clinical models were established by the same feature selection and machine learning (ML) pipeline as TTF2Pred. Median values over repetitions (CV) and bootstrapping (validation) are indicated. (**D**) Confusion matrices for training, CV, and independent validation of the best multiparameter Clin model and CA19-9 for S- and L-TTF2 prediction. Rows: Actual class. Columns: Predicted class. (**E**) Calibration curves for L-TTF2 prediction (positive class) following CV and validation, comparing TTF2Pred performance to an ideal reference line. Curves with 95% confidence intervals were generated using sigmoidal 4-parameter (4PL) least squares fitting over CV repetitions and bootstrapping for validation. (**F**) Predictive performance of the ZeroR majority class baseline classifier. Accuracy and ROC-AUC remained constant across feature models and for both TTF2 and OS binary class validation sets. The number of patients per S-/L-TTF2 group in training and validation sets are indicated in Fig. 3D. *****P*≤0.0001.

Fig. S10

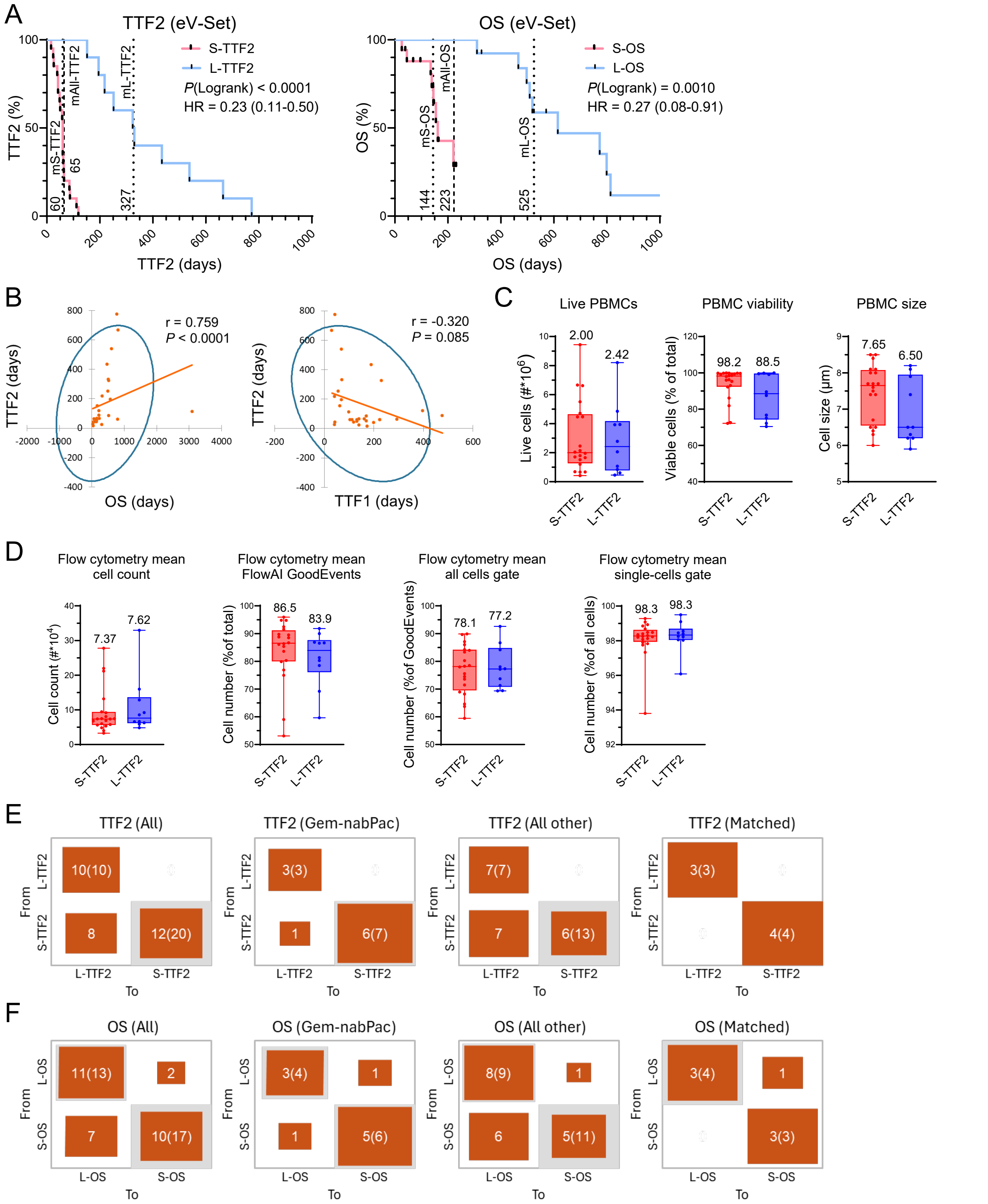

Fig. S10: TTF2Pred has prognostic value and predicts second-line chemotherapy outcomes across therapy regimens. (**A**) Kaplan-Meier survival curves for TTF2 and OS in S- vs. L-TTF2 and S- vs. L-OS groups from the external validation cohort (eV-Set, *n*=30). Log-rank P-values, hazard ratios (HR, with 95% confidence interval), and median TTF2/OS values for each cohort and the combined group are shown. (**B**) Spearman correlation of TTF2 with overall survival (OS) from the start of second-line treatment and first-line treatment failure (TTF1) in the eV-Set PBMC analysis cohort. (**C-D**) Quantification and quality control of PBMC samples from S- and L-TTF2 patients in the eV-Set PBMC analysis cohort. Median values are indicated, respectively. (**C**) Overall PBMC yield and viability, including total live PBMC count, viability percentage, and average PBMC size. (**D**) Protein expression profiling (PPEP) quantity assessment, including post-flow cytometry cell count, percentage of retained cells after automated cleaning with FlowAI, and percentage of cells in key pre-gating steps (all cells, single-cells), shown relative to the respective parental gate. All PPEP data represent means across eight flow cytometry subpanels. (**E-F**) Confusion matrices for independent validation of the eV-Set subcohorts defined based on the applied first-line therapy (Fig. 5H) using the TTF2Pred model for (**E**) S-/L-TTF2 (predictive performance) and (**F**) S-/L-OS (prognostic performance) binary classification. Rows indicate actual class, and columns indicate predicted class. The number of patients per eV-Set subcohorts is indicated in Fig. 5H.

Fig. S11

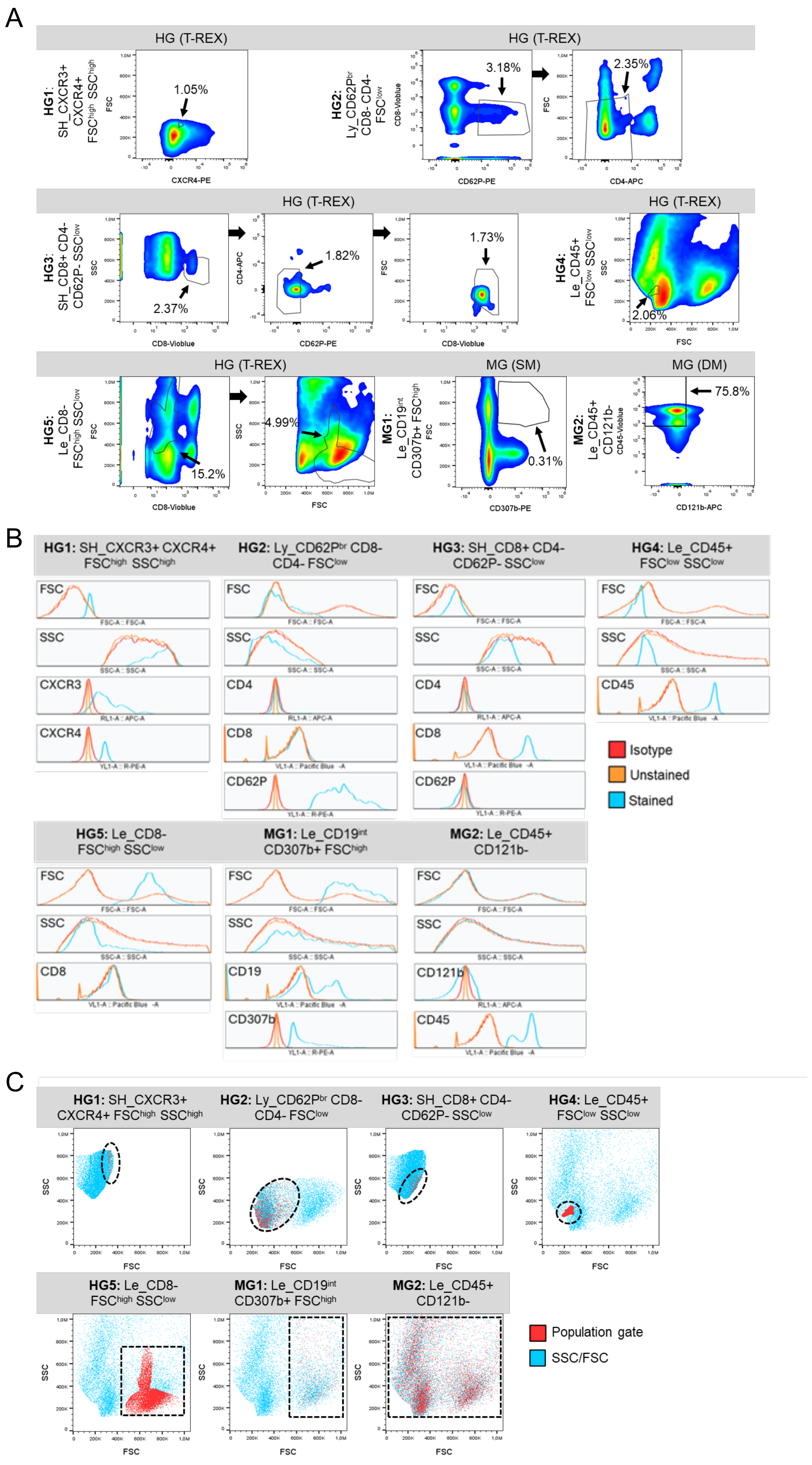
A
HG (T-REX)
HG1: SH_CXCR3+ CXCR4+ FSChigh SSChigh
1.05%
CXCR4-PE
HG2: Ly_CD62Pbr CD8- CD4- FSClow
3.18%
2.35%
CD62P-PE
CD4-APC
HG3: SH_CD8+ CD4- CD62P- SSClow
2.37%
1.82%
1.73%
CD8-Vioblue
CD62P-PE
HG4: Le_CD45+ FSClow SSClow
2.06%
FSC
HG5: Le_CD8- FSChigh SSClow
15.2%
4.99%
MG (SM)
MG1: Le_CD19int CD307b+ FSChigh
0.31%
CD307b-PE
MG (DM)
MG2: Le_CD45+ CD121b-
75.8%
CD121b-APC
B
HG1: SH_CXCR3+ CXCR4+ FSChigh SSChigh
HG2: Ly_CD62Pbr CD8- CD4- FSClow
HG3: SH_CD8+ CD4- CD62P- SSClow
HG4: Le_CD45+ FSClow SSClow
FSC
SSC
CXCR3
CXCR4
CD4
CD8
CD62P
CD45
Isotype
Unstained
Stained
HG5: Le_CD8- FSChigh SSClow
MG1: Le_CD19int CD307b+ FSChigh
MG2: Le_CD45+ CD121b-
CD19
CD307b
CD121b
C
Population gate
SSC/FSC

Fig. S11

Fig. S11: TTF2Pred model features are characterized by protein markers, cell size, and cell granularity. (**A-C**) Molecular characterization of the TTF2Pred model features (PBMC immune populations) including five automated clustering-based HyperGates (HG1-5) and two manually established ManualGates (MG1-2). Data represent concatenated single-cell events from flow cytometry across all 82 patients in the PREDICT PBMC cohort. During concatenation, events were down-sampled to ensure equal representation across all patients. (**A**) Flow cytometry gating strategy for TTF2Pred PBMC immune populations. Gates are indicated by arrows, with corresponding nomenclature. The population frequency, expressed as a percentage of the respective CD45 HG across all 82 patients (concatenated events), is provided. MG1 was defined based on a single marker (SM), while MG2 incorporated a dual-marker (DM) gating strategy. (**B**) Flow cytometry histograms of relevant markers included in TTF2Pred PBMC immune populations and initially selected based on NanoString tumor tissue profiling (Fig. S5C). Unstained and isotype control histograms from the corresponding CD45 HyperGate population were used to validate marker positivity. (**C**) SSC (side scatter) vs. FSC (forward scatter) back-gating for TTF2Pred PBMC immune populations. Defined population gates are highlighted by dotted lines within the SSC/FSC window. Br: Bright fluorescence intensity; Int: Intermediate fluorescence intensity; Le: Leukocytes; Ly: Lymphocytes; SH: SSC-high.

Fig. S12

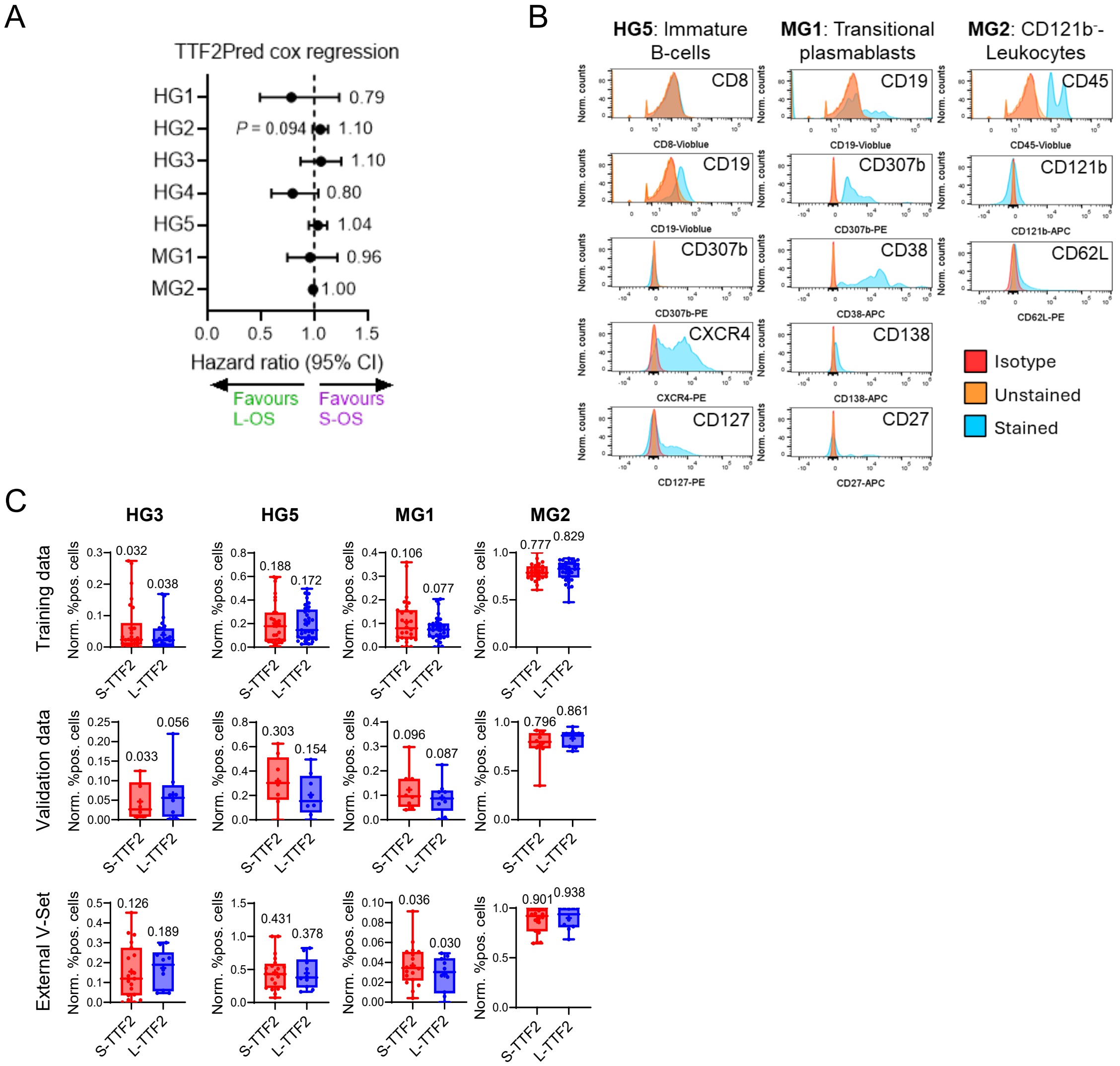

Fig. S12: Circulating PBMC subpopulations defining the predictive TTF2Pred immune signature. (**A**) Cox multivariate regression analysis of individual TTF2Pred features associated with overall survival (OS) from the start of second-line treatment in the PREDICT PBMC cohort (*n*=82). Hazard ratios (HR) with 95% confidence intervals (CI) are shown; median HR values are indicated. Significant markers are highlighted in green (favorable, associated with long-TTF2) or purple (unfavorable, associated with short-TTF2). (**B**) Flow cytometry characterization of additional PBMC protein markers to further refine the TTF2Pred gates HG5 and MG1-2, with corresponding putative immune subsets indicated. Unstained and isotype controls were used to validate marker positivity. Data represent concatenated single-cell events from *n*=4 representative PREDICT PBMC cohort patients. During concatenation, events were down-sampled to equalize patient representation. (**C**) Min-max-normalized (Norm.; 0-1) frequencies of TTF2Pred immune subsets (HG3, HG5, MG1-2) in S- and L-TTF2 groups across PREDICT (PBMC cohort) training (S-TTF2: *n*=32; L-TTF2: *n*=34), validation (S-TTF2: *n*=8; L-TTF2: *n*=8), and external validation (eV-Set; S-TTF2: *n*=10; L-TTF2: *n*=20) cohorts. Median values are indicated.

Fig. S13

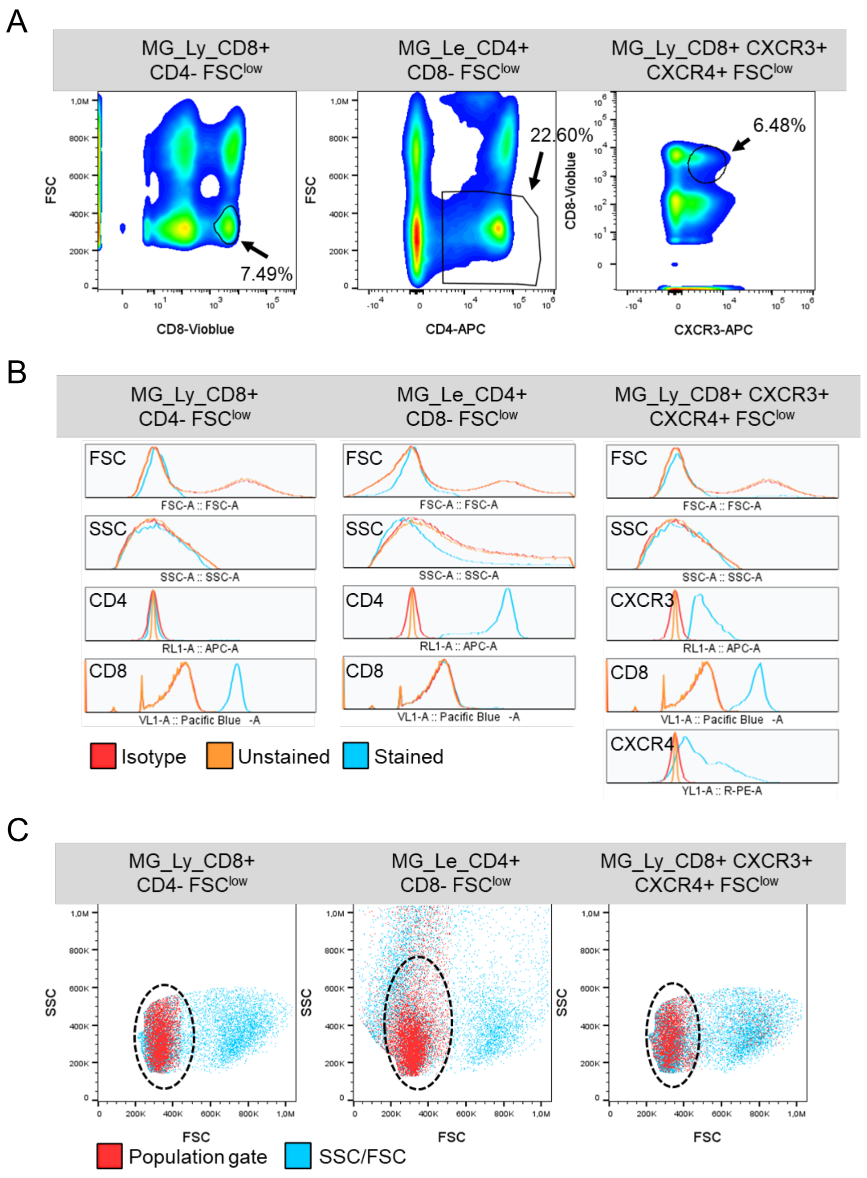

Fig. S13: The highest ranked PBMC subsets identified by statistical filtering mirror the treatment-naïve tumor immune microenvironment (TiME) in peripheral blood after one therapy line. (**A-C**) Molecular characterization of the top three ranked features from the Significance method using the combined feature list (PPEP: PMC protein expression profiling; PGEP: PBMC gene expression profiling; Clin: all available clinical data) as input. All three features represented ManualGates (MGs). Data represent concatenated single-cell events from flow cytometry across 82 patients in the PREDICT PBMC analysis cohort. During concatenation, events were down-sampled to ensure equal representation across all patients. (**A**) Flow cytometry gating strategy for the PBMC immune populations. Gates are indicated by arrows, with corresponding nomenclature. The population frequency, expressed as a percentage of the respective CD45 HG across 82 PREDICT patients (concatenated events), is provided. (**B**) Flow cytometry histograms of relevant protein markers included in the respective PBMC immune populations and initially selected based on NanoString tumor tissue profiling (Fig. S5C). Unstained and isotype control histograms from the corresponding CD45 HyperGate population were used to validate marker positivity. (**C**) SSC (side scatter) vs. FSC (forward scatter) back-gating for respective PBMC immune subpopulations. Defined population gates are highlighted by dotted lines within the SSC/FSC window. Le: Leukocytes; Ly: Lymphocytes; SH: SSC-high cells.
