# Supplementary material for: An immune responsive tumor microenvironment imprints into PBMCs and predicts outcome in advanced pancreatic cancer: lessons from the PREDICT trial
Source: Mol Cancer. 2025 Jul 22;24:202. doi: 10.1186/s12943-025-02406-7 (PMC12281745; doi:10.1186/s12943-025-02406-7)
Supplement: Supplementary file 3 — Additional file 3. Supplementary methods supporting the data presented in the main text [file 12943_2025_2406_MOESM3_ESM.docx]

**Supplementary Methods and Materials**

**FFPE tissue laser capture microdissection, RNA extraction, and RNA quantification**

FFPE tissues were obtained from treatment-naïve PREDICT tissue cohort patients at diagnosis (Fig. 1A) and extracted either during surgical tumor resection or via fine-needle biopsy. Tissues were stored at 4°C with <50% relative humidity. Laser capture microdissection (LCM) was applied to FFPE sections to isolate regions with comparable tumor and non-tumor area percentages between all analyzed samples. For RNA extraction, no fewer than six consecutive 10 µm-thick FFPE sections of each sample were pooled, deparaffinized, and digested with proteinase K overnight. Subsequently, nucleic acids were extracted automatically using a Maxwell 16 Research system and the RNA FFPE Purification Kit (all Promega, Madison, Wisconsin). After purification, nucleic acids were treated with DNase I at room temperature for 15 min and subsequently used for RNA-based targeted gene expression profiling. The concentration of RNA was measured fluorometrically using the Qubit HS RNA assay kit (Thermo Fisher Scientific, Waltham, Massachusetts). All assays were performed according to the manufacturers’ protocols and as previously described [1].

**Targeted gene expression profiling (NanoString)**

Targeted mRNA expression profiling was conducted on the NanoString nCounter gene expression platform (NanoString Technologies, Seattle, Washington) using a 770-gene panel (PanCancer Human IO 360 Panel; further information: <https://nanostring.com/products/ncounter-assays-panels/oncology/pancancer-io-360/>). Immune gene signatures and expression profiling were performed as previously published [2]. Statistical analysis and graphics generation were performed using the programming language R. Expression data were preprocessed by background subtraction and subsequent sample normalization. For sample normalization, the 20 panel genes with the lowest coefficient of variation and an expression level of at least 100 in the TCGA-PAAD dataset (bulk RNA-seq; n=179; <https://portal.gdc.cancer.gov/projects/TCGA-PAAD>, accessed 20 April 2025) [3] were used as housekeepers. The gene expression profile of each sample was scaled by the median expression level of these housekeeping genes. Gene expression data were log_2_-transformed prior to statistical analysis and the mean log_2_ fold change was calculated for group comparison (Table S3).

**Transcriptome exploratory data analysis**

Gene expression analysis was initiated by removing outliers from log_2_-transformed data using the ROUT method (Q=2%) in GraphPad Prism v10.4.1 (RRID:SCR_002798) and genes were retained only if ≥50% of data points per S-/L-TTF2 group were valid. Statistical comparison between groups was performed using ordinary two-way ANOVA followed by Sidak’s post-hoc multiple comparison test, implemented in GraphPad Prism (Table S3). Principal component analysis (PCA) for patients (observations) was conducted using the ClustVis webtool (<https://biit.cs.ut.ee/clustvis/>) [4] via singular value decomposition (SVD) with imputation after unit variance scaling. Gene (variable) correlation biplots were generated through Pearson correlation-based PCA with k-nearest neighbor (KNN) imputation in XLSTAT v2024.4 (RRID:SCR_016299). Hierarchical clustering was performed using ClustVis (RRID:SCR_017133) [4] and included unit variance-scaling of rows, correlation (rows) and binary (columns) distance calculation via average linkage, and missing value imputation. Functional annotation of gene clusters was achieved through overrepresentation analysis (ORA) of GO Molecular Functions (GOMF), GO Biological Processes (GOBP), and KEGG gene set collections using the ShinyGO webtool with FDR cut-off < 0.05 (RRID:SCR_019213; <http://bioinformatics.sdstate.edu/go/>) [5]. Gene Set Enrichment Analysis (GSEA) of MSigDB Canonical Pathways (CP; <https://www.gsea-msigdb.org/gsea/msigdb/human/genesets.jsp>), TISIDB immune signatures (RRID:SCR_018821; <http://cis.hku.hk/TISIDB/>; Table S4) [6], and PdacR PDAC subtype signatures (<http://pdacR.bmi.stonybrook.edu>; Table S4) [7] was executed in GSEA v4.3.2 (RRID:SCR_003199) with weighted enrichment statistics and Signal2Noise gene ranking (cut-offs for the global analysis: p < 0.05 and FDR < 0.2; the FDR cut-off chosen following MSigDB recommendations). Immune profiling incorporated an 18-gene tumor inflammation panel and immune infiltration signatures provided by the NanoString PanCancer IO 360 Panel (Table S4). TCGA-PAAD data were mined with GEPIA2 (RRID:SCR_026154; <http://gepia2.cancer-pku.cn/#index>) [8] using ANOVA and Spearman correlation, complemented by immune signature interrogation with TISIDB [6]. Single-cell RNA-seq data GSE154778 (n=16; <https://www.ncbi.nlm.nih.gov/geo/query/acc.cgi?acc=GSE154778>, accessed 24 April 2025) were mined using the TISCH2 webtool (RRID:SCR_018821; <http://tisch.compbio.cn/>) [9] with mean-based gene collapse. The STITCH v5.0 (STRING v10) webtool (RRID:SCR_007947; <http://stitch.embl.de/cgi/input.pl?UserId=dYuzAsrnRoNV&sessionId=8fsvnpaRvYel>) [10] was used to reconstruct molecular interaction networks through integration of active interaction sources, text mining, experimental data, databases, gene neighborhoods, gene fusions, and co-expression patterns. PBMC-specific single-cell RNA-seq data were mined using the THPA webtool (RRID:SCR_006710; <https://www.proteinatlas.org/>; single-cell > tissues > PBMC) [11].

**Multiplexed immunofluorescence (mIF) histological staining**

The mIF procedure was carried out using the Opal multiplex system (Akoya Biosciences, Marlborough, Massachusetts) following the manufacturer's instructions. Briefly, FFPE sections were deparaffinized, fixed with 4% paraformaldehyde, and subjected to antigen retrieval using Tris/EDTA (pH 9) for heat-induced epitope retrieval at 95°C for 15 min. Each section underwent multiple rounds of staining with one round including endogenous peroxidase blocking, protein blocking, primary antibodies (Table S1), and the corresponding secondary horseradish peroxidase-conjugated polymer (Akoya Biosciences, Marlborough, Massachusetts). Tyramide signal amplification was employed to bind each horseradish peroxidase-conjugated polymer with different fluorophores (Table S1). The bound antibodies were removed after antigen retrieval for the next round of staining. Following all sequential staining reactions, sections were counterstained with DAPI (Vector Laboratories, Newark, California). Using the Zeiss Axio Scanner Z.1 (Carl Zeiss AG, Oberkochen, Germany) at 10× objective magnification, slides were scanned and digitized. Quantification of individual and/or co-expressing markers in the multiplexed immunofluorescence images was performed using the HALO image analysis software (Indica Labs, Albuquerque, New Mexico; RRID:SCR_018350) as previously described [12]. Briefly, a gate for region of interest (ROI) was manually drawn to mark tumorous tissues on the image of each slide. Areas containing tissue folds and stain artifacts were manually excluded. Tissue classification was performed to identify tumor and stroma regions within the ROIs. Nuclear detection was performed based on DAPI staining. Signal intensity for positivity of each marker was manually adjusted and the same signal thresholds for positivity were applied to all tissues within the cohort.

**PBMC isolation from EDTA-anticoagulated blood**

All peripheral blood mononuclear cells (PBMCs) analyzed in this study were collected from patients directly prior to the start of second-line treatment (Fig. 1A). PBMCs were isolated from one vial of EDTA-anticoagulated blood per patient using density gradient centrifugation. Blood samples were diluted 1:1 with PBS (Gibco, Waltham, Massachusetts; #14200067) and carefully layered onto Pancoll separation medium (PAN-Biotech, Aidenbach, Germany; #P04-60500) in a 1:1 ratio. The gradient was centrifuged at 800 × g for 20 min at room temperature with minimal acceleration and deceleration. The mononuclear cell layer was carefully harvested and washed twice with PBS at 311 × g for 7 min. Isolated PBMCs were resuspended in cold RPMI-1640 medium (Gibco, Waltham, Massachusetts; #21875-034) supplemented at a 1:1 ratio with freezing medium consisting of 80% FBS Superior (Biochrom, Berlin, Germany; #S0615) and 20% DMSO (Sigma-Aldrich, St. Louis, Missouri, #D2650). Cell suspensions were aliquoted and cryopreserved using controlled-rate freezing (-1°C/min) in a Mr. Frosty container (Thermo Scientific, Waltham, Massachusetts) at -80°C before long-term storage in liquid nitrogen (-196°C).

**PBMC protein expression profiling (PPEP)**

PBMC protein expression profiling (PPEP) was the investigation of PBMC surface markers at the protein level based on flow cytometry and two distinct gating procedures for data analysis (MG: ManualGating, HG: HyperGating).

**PPEP - Flow cytometry**

Frozen PBMCs (liquid nitrogen) were rapidly thawed in a 37°C water bath and slowly diluted with pre-warmed RPMI medium 1640 (Gibco, Waltham, Massachusetts; #21875034) containing 20% FBS (Gibco, Waltham, Massachusetts; #10270106). After gentle resuspension, cells were centrifuged at 350 × g for 8 min at 4°C and subsequently resuspended in FACS buffer (DPBS pH 7.2; 0.5% BSA, Sigma-Aldrich, St. Louis, Missouri, #A9418; 2 mM EDTA, AppliChem, Darmstadt, Germany, #A3145). Using an automated cell counter (CytoSMART, Corning, New York) with trypan blue staining (Gibco, Waltham, Massachusetts, #11538886; 1:1 dilution), cells were counted and cell viability as well as cell size were determined. Flow cytometry was performed as previously described by Lahusen *et al.* [13]. In brief, counted cells were washed in FACS buffer via centrifugation and aliquoted into a 96-well U-bottom plate (Corning, New York) with 2×10^5^ cells/well. Cells on the plate were washed and stained with 1 µL of respective antibodies (Table S1; Miltenyi Biotec, Bergisch Gladbach, Germany) in 100 µL FACS buffer for 10 min at 4°C in the dark. After washing and resuspension in FACS buffer (250 µL/well), samples were analyzed on an Attune NxT flow cytometer (Thermo Fisher Scientific, Waltham, Massachusetts). The protocol aimed to collect a maximum of 25 000 events in the single-cell gate, with samples measured at a flow rate of 500 µL/min. The eight flow cytometry staining subpanels used for gate (feature) selection with the corresponding functional subgroup annotations are shown in Table S6.

**PPEP - Pre-processing and pre-gating**

Data analysis was performed using FlowJo v10.8.1 (RRID:SCR_008520, <https://www.flowjo.com/>, BD) for Windows 11 v24H2 (Microsoft) in combination with R v4.3.3 (RRID:SCR_001905, <https://www.R-project.org/>) for Windows 11 v24H2. Flow cytometry data analysis followed a systematic pipeline (Fig. 3C, S6A). Data were initially transferred to the FlowJo software, where samples were labeled according to TTF2 group membership (S-TTF2 vs. L-TTF2, Fig. 3A). FlowAI v2.3.2 [14] was then employed for quality control using all relevant flow cytometry parameters to exclude anomalies, including the evaluation of flow rate, signal acquisition, and dynamic range. A pre-gating strategy was implemented by removing margin events from all events and performing single-cell selection based on forward scatter (FSC)-height (H)/FSC-area (A) flow parameters (Fig. S6A-B). The HyperFinder v0.8.1 algorithm (<https://www.flowjo.com/exchange/#/plugin/profile?id=27>, based on GateFinder [15] and Hypergate [16]), which uses machine learning (ML) to generate an optimal gating sequence and gate shapes for any population of interest, was applied to back-gate CD45-positive (+) cells (leukocytes, Le), CD45+ side scatter (SSC)-low cells (lymphocytes, Ly), and CD45+ SSC-high cells (SH cells) to the SSC/FSC window using the flow cytometry staining subpanel P7 that included CD45-Vioblue (Fig. S6C, Table S6). This enabled the transfer of these three specific leukocyte subpopulations/CD45 HyperGates (HGs) to the single-cell gates of all other flow cytometry subpanels for further analysis (Fig. S6B). The final step involved the concatenation of events per leukocyte subpopulation (Le, Ly, SH cells; Fig. S6B) over all samples used for gate selection (40 patients with longest/shortest TTF2 and matching TTF2 as well as OS short/long groups). Concatenation was applied with down-sampling (DownSample v3.3.1: <https://www.flowjo.com/exchange/#/plugin/profile?id=25>) to the minimal number of events identified across all samples (same number of events per TTF2 group) per leukocyte subpopulation and per flow cytometry subpanel. For group separation after concatenation, gates were automatically established on count/group peaks, with each group representing exactly 50% of the total number of events (Fig. S6A). These concatenated populations were used for ManualGating (MG) and HyperGating (HG) procedures, respectively.

**PPEP - ManualGating (MG)**

The MG/classical gating procedure (Fig. 3C) was conducted using unstained and isotype controls as reference points to draw gates on distinct fluorescence-positive (+) populations. Single- and double-marker-positive (+) populations, as well as distinct SSC or FSC populations combined with a marker, were included during MG across all leukocyte subpopulations and subpanels. Depending on the markers included per subpanel (Table S6), the final step involved transferring all manually drawn gates per leukocyte population to markers representing distinct immune cell subpopulations. These included CD8+ (CD8^+^-T-cells), CD19+ (B-cells), CD45+ (all leukocytes), and NKG2D+ (NK-cells) cells.

**PPEP - HyperGating (HG)**

The HG procedure was initiated by clustering of single-cell events using three different algorithms separately: KNN-based density clustering via xShift v1.4.1 [17], self-organizing maps (SOM) with two-level clustering via FlowSOM v4.1.0 [18], and UMAP/KNN-based clustering for the identification of differences between two groups was conducted using T-REX v1.2.2 (Fig. S6A) [19]. All algorithms were employed with FlowJo standard settings and utilized all relevant flow cytometry parameters (fluorescence per marker, FSC, SSC). The algorithm xShift was set to automatic K optimization, resulting in an optimized number of clusters, which was then used as the target cluster number for the FlowSOM algorithm. The T-REX algorithm was applied to identify the most distinct clusters for S-TTF2 vs. L-TTF2 dichotomous comparison, with bins set to ≥ 65% and ≤ 35%. Only T-REX clusters containing single-cell event numbers ≥0.1% of all concatenated events per subpanel and leukocyte subpopulation were included. Marker enrichment modeling (MEM) [20] was used to quantify marker enrichment between single-cell clusters by comparing expression patterns and generating interpretable scores from -10 to +10. MEM was applied separately on fluorescence parameters and FSC/SSC parameters for cluster comparison and marker annotation. ClusterExplorer v1.7.6 (<https://www.flowjo.com/exchange/#/plugin/profile?id=30>) with inclusion of annotations for the percentage distribution of clusters in S-TTF2 vs. L-TTF2 groups was used for cluster selection (Fig. S6A). All clusters showing at least a 10% difference between S-/L-TTF2 groups (%L-TTF2 < 45 or > 55) were manually selected. The MEM algorithm [20] was then applied again to generate merged single-cell clusters, carried out separately for fluorescence-based clusters and those based solely on FSC/SSC parameters. The algorithm utilized MEM scores with a threshold set to ≥1 to identify and combine similar clusters. Finally, to generate optimal gating sequences and gate shapes from all selected single-cell clusters, the machine learning-based HyperFinder function was used. In short, HyperFinder uses ML by division of input data into training and validation sets with iterative adjustment of gate shapes and numbers to optimize the F-measure score. Both polytope and polygon algorithms were tested with gate boundary optimization, and the corresponding gate with the highest F-value was selected for each case. Further information and availability of the algorithms used for flow cytometry data analysis can be found at the FlowJo Exchange website (<https://www.flowjo.com/exchange/#/>).

**PPEP - Final gates transfer and nomenclature**

The pre-established gates were subsequently applied to all analyzed patient samples from different cohorts, following the comprehensive pre-gating protocol as described above (Fig. S6A). This protocol was executed sequentially, beginning with data labeling (S-/L-TTF2). Anomalies were then eliminated using FlowAI, followed by the implementation of an all-cells gate to exclude margin events and subsequently a single-cell gate. Leukocyte subpopulations were then delineated using CD45 HyperGates, categorizing cells into leukocytes (Le), lymphocytes (Ly), and SSC-high (SH) populations (Fig. S6B-C). Finally, both ManualGates (MGs) and HyperGates (HGs) were employed for each CD45 HG (leukocyte) subpopulation and flow cytometry subpanel separately, thereby completing the gating strategy (Fig. S6A). Relevant HGs and MGs are described by a specific nomenclature shown in Fig. S6D.

**PBMC gene expression profiling (PGEP)**

PBMC gene expression profiling (PGEP) was the analysis of PBMC intracellular markers at the gene expression level based on RT-qPCR and relative or fold change (to immune markers) data analysis.

**PGEP - RT-qPCR**

The residual PBMCs following flow cytometry analysis (PPEP) were washed with DPBS (Gibco Waltham, Massachusetts). Total RNA extraction was then performed using the RNeasy Mini kit (Qiagen, Hilden, Germany, #74104), incorporating on-column DNase digestion to ensure RNA purity. The extracted RNA was reverse transcribed to complementary DNA (cDNA) using the iScript cDNA Synthesis Kit (Bio-Rad, Hercules, California, #1708890), strictly following the manufacturer's protocols. Quantitative reverse transcription polymerase chain reaction (qRT-PCR, RT-qPCR) was conducted using PowerUP SYBR Green Master Mix (Applied Biosystems, Waltham, Massachusetts, #A25742) in conjunction with QuantiTect Primer Assays (Qiagen, Hilden, Germany, #24990) on the Quantstudio 3 Real-Time PCR System (Applied Biosystems, Waltham, Massachusetts, RRID:SCR_018712). The specific primer sequences used in this study are detailed in Table S1.

**PGEP - Data analysis and nomenclature**

RT-qPCR data analysis followed a systematic pipeline (Fig. 3C) and was performed using Excel v16.0 (RRID:SCR_016137, Microsoft) on Windows 11 v24H2. First, relative expression was determined for each marker gene using the 2^(-ΔCT) method, with *GAPDH* serving as the endogenous reference (housekeeping) gene for normalization. Next, fold changes (ratios) were calculated for each gene with marker genes representing distinct immune cell subpopulations including *PTPRC* (leukocytes), *CD8A* (CD8^+^-T-cells), *CD19* (B-cells), and *NCR1* (NK-cells). Fold changes were calculated using the 2^(-ΔΔCT) method [21]. Relevant genes (relative expression) or ratios (fold change to standard immune markers) are described by a specific nomenclature shown in Fig. S6D.

**Machine learning (ML)**

To identify a minimally predictive biomarker signature for short-/long-TTF2 and short-/long-OS classification, we have chosen to utilize supervised machine learning (ML) due to our large feature space. For feature matrices >2000 features, full-feature Cox regression may suffer from high dimensionality and multicollinearity, forcing drastic pre-filtering or penalization that can obscure discriminative markers. A full feature Cox regression would also not yield a minimal biomarker signature. Therefore, we opted for a combination of rigorous feature selection pipelines and supervised ML.

Thus, a comprehensive ML classification methodology was implemented for binary classification tasks using the Java-based Weka v3.8.6 environment [22] (RRID:SCR_001214, University of Waikato, <https://ml.cms.waikato.ac.nz/weka/>, Fig. 3D-E). Experimenter and KnowledgeFlow environments were used. All algorithms were used at Weka standard settings unless stated otherwise.

**ML - Pre-processing and data partitioning**

The initial data pre-processing (filtering) phase included unsupervised feature (attribute) normalization (attribute.Normalize) and patient (instance) randomization (instance.Randomize) to eliminate order bias. The PREDICT dataset was then divided into a training set (T-Set, *n*=66) and a validation set (V-Set, *n*=16) through random sampling (instance.RemovePercentage, percentage=80), maintaining an 80%/20% T-/V-Set split ratio (Fig. 3D). The class balance was preserved between both sets in the PREDICT cohort. The T-Set contained 34 L-TTF2 and 32 S-TTF2 samples, while the V-Set for both TTF2 and OS binary classes had an equal distribution with 8 samples per group. The external independent cohort (eV-Set) was unbalanced, with fewer patients in the long (L-TTF2: *n*=10, L-OS: *n*=13) and more patients in the short (S-TTF2: *n*=20, S-OS: *n*=17) cohort (Fig. 5F). The eV-Set was further separated into subcohorts based on the first-line chemotherapy (CTX) received as shown in Fig. 5H: All first-line treatments vs. first-line Gem-nabPac only vs. all other first-line treatments other than Gem-nabPac as shown in Fig. 5G vs. first-line Gem-nabPac and second-line Nal-IRI/5FU/LV exactly matching the PREDICT trial treatment.

**ML - Minimal feature combination selection and optimization**

Feature selection was conducted using significance-based, wrapper-based, and combined (hybrid) approaches on the internal T-Set to identify the optimal minimal feature subset (Fig. 3E). The significance-based approach utilized the Weka-based SignificanceAttributeEval algorithm with Ranker as the search method, evaluating ranked attribute worth by statistical significance-based on Chi-squared (categorial) and ANOVA (continuous) tests applied per feature. This method included selecting the top three or five (Sign Top5 signature) features and PCA-based ranking. PCA-based ranking was performed plotting PCA in Addinsoft XLSTAT v2024.4 (RRID:SCR_016299, <https://www.xlstat.com/de/>, used with Microsoft Excel v16.0.) and selecting features with significant squared cosines in the PCA F-axis with the highest PCA Eigenvalue (F1-axis; Sign PCA topF1 signature). The wrapper method employed the Weka-based WrapperSubsetEval algorithm with eight carefully selected classification algorithms (Fig. 3D-E) which showed best performance for this specific dataset: NaïveBayes (NB), KernelLogisticRegression (KLR), Logistic (logistic regression, LR), SMO (sequential minimal optimization), IBk (instance-based KNN), RandomTree (RT), RandomForest (RF), and J48 (C4.5 tree). The wrapper method evaluated attributes through automated classifier learning using cross-validation (CV) evaluation loops. The wrapper search method was bi-directional BestFirst with searchTermination set to 20 for all classifiers except RF, which had searchTermination set to 5 to reduce calculation/running time. In the combined (hybrid) approach, only features previously ranked among the top features from the significance-based method with a rank score cut-off > 0.5 were used as input for the wrapper method (Sign Wrapper signature; Fig. 3E). After using multiple feature input combinations for the wrapper method and the feature panels from significance and hybrid approaches (Fig. 4A), the overall best performing and most minimal feature subsets with the corresponding classifier were selected by comparing accuracies and ROC-AUCs from 10x 10-fold CV. In the case of similar performances between different subset/classifier combinations (Fig. S8A), the subset with the fewest features was selected (minimal signature; Fig. S8B). Selected best performing subset/classifier combinations were then optimized through iterative trimming (removal) of non-informative features (ranking ≤ 0) using the following Weka-based feature evaluation methods: ClassifierAttributeEval, CVAttributeEval, InfoGainAttributeEval, and ReliefFAttributeEval. This was followed by parameter optimization via CVParameterSelection and missing value replacement (features) via attribute.ReplaceMissingValues where beneficial for the performance. This exact same procedure described above was applied for all available clinical data (Clin) and CA19-9 only to generate similarly optimized and comparable clinical benchmark minimal models (Fig. 4B).

**ML - Performance analysis**

Bootstrapping was used to measure training performance in the T-Set (PREDICT cohort TTF2) and validation performance in the independent V-Set (PREDICT internal cohort TTF2/OS, external cohort TTF2/OS). For bootstrapping (10x with replacement), the instance.Resample filter in Weka was repeatedly applied to the T-Set with ten different randomly generated seed numbers using the Data sampling function (random with replacement) in XLSTAT v2024.4 in Excel v16.0. Further, performance and robustness of subset/classifier combinations for the PREDICT cohort T-Set were evaluated using 10x 10-fold CV via CrossValidationFoldMaker in Weka with ten randomly generated numbers (Data sampling with XLSTAT v2024.4 in Excel v16.0) for the random seed (Fig. 3D). Validation cohorts remained unchanged after partitioning and across all performance metrics. The Weka performance metrics used included: True positive rate (TPR), false positive rate (FPR), ROC curve, ROC-AUC value, Accuracy, Specificity, Sensitivity, Precision, and classifier prediction scores. Furthermore, the Youden Index (J) [23] was calculated based on Sensitivity and Specificity. All performance metrics were calculated for the optimal probability threshold defined by maximized Youden Index (J). The positive class for ROC-AUC curves was L-TTF2. Additional Weka performance metrics used for evaluation of the imbalanced external cohort (eV-Set), included PRC-AUC and Matthews Correlation Coefficient (MCC) [24] scores. The ZeroR classifier in Weka, which predicts the majority class (for categorical targets) or the mean value (for numeric targets) by ignoring all predictors and relying solely on the target variable's distribution, was used as the baseline ML performance control. Confusion matrices were plotted using the Model Performance Indicators function in XLSTAT v2024.4 in Excel v16.0. Calibration curve raw data were generated with Weka's CalibrationCurve plugin. Final sigmoidal calibration curves were generated using nonlinear least squares regression to fit a 4-parameter logistic model (4PL, outliers to Q=2% eliminated, no weighting) in GraphPad Prism v10.4.1 (RRID:SCR_002798, <https://www.graphpad.com/>). Further information about used Weka algorithms can be found at the Weka documentation website from the University of Waikato: <https://weka.sourceforge.io/doc.dev/overview-summary.html>.

**Blood smear Wright-Giemsa staining**

Blood smears were prepared on Superfrost microscope slides (Thermo Scientific, Waltham, Massachusetts) and stained with RAL Diff-Quik (Siemens Healthineers, Erlangen, Germany; modified Wright-Giemsa), per manufacturer’s protocol. Slides were coverslipped with Entellan (Merck, Darmstadt, Germany) and examined at 60× magnification with immersion oil using the BZ-X Series microscope (Keyence, Osaka, Japan; RRID:SCR_016979).

**Effect-size estimation and power assessment**

To validate the appropriate cohort size for our NanoString IO360 analysis (*n*=10 per outcome group), we performed an initial effect-size and power analysis. The effect size was calculated as Cohen’s d=(μ1-μ2)/σ, where μ1 and μ2 are the mean log₂-normalized counts for the investigated groups, respectively, and σ is the pooled standard deviation. NanoString data in general is highly reproducible and shows low technical noise. Thus, recent immune oncology studies using the IO360 panel demonstrated strong expression differences, which typically yielded: Log_2_-fold changes (log_2_FC)≥1 with adjusted p-values (FDR)<0.05 as well as technical standard deviations of ~0.6-0.8 log₂ units for moderately expressed genes [25,26]. Consequently, a two-fold change (log₂FC=1) corresponds to a large effect size (d≈1.2-1.5). Power was calculated with a two-sample, two-sided t-test (α=0.05) using G*Power 3.1 (version 3.1; RRID: SCR_013726) [27]. With *n*=10 per group the achievable power is 0.88 (β=0.12) for d=1.2, 0.74 (β=0.26) for d=1.0, and 0.56 (β=0.44) for d=0.8. Thus, the study is well powered to detect strong expression differences (log₂FC≥1, d≥1.0) in line with our aim to identify biomarkers that clearly separate the S- and L-TTF2 groups. Moderate changes (d≈0.8) carry a higher risk for false negatives. We therefore focused our downstream analyses and biological interpretation on genes displaying d≥1.0. Cohen’s d values for the most relevant NanoString differentially expressed genes in our study were calculated as described above using α=0.05 and two-sided unpaired t-test (Table S3).

**References**

1. Kirchner M, Neumann O, Volckmar A-L, Stögbauer F, Allgäuer M, Kazdal D, et al. RNA-Based Detection of Gene Fusions in Formalin-Fixed and Paraffin-Embedded Solid Cancer Samples. Cancers. 2019;11:1309.

2. Budczies J, Kirchner M, Kluck K, Kazdal D, Glade J, Allgäuer M, et al. A gene expression signature associated with B cells predicts benefit from immune checkpoint blockade in lung adenocarcinoma. OncoImmunology. 2021;10:1860586.

3. Raphael BJ, Hruban RH, Aguirre AJ, Moffitt RA, Yeh JJ, Stewart C, et al. Integrated Genomic Characterization of Pancreatic Ductal Adenocarcinoma. Cancer Cell. 2017;32:185-203.e13.

4. Metsalu T, Vilo J. ClustVis: a web tool for visualizing clustering of multivariate data using Principal Component Analysis and heatmap. Nucleic Acids Res. 2015;43:W566–70.

5. Ge SX, Jung D, Yao R. ShinyGO: a graphical gene-set enrichment tool for animals and plants. Bioinformatics. 2019;36:2628–9.

6. Ru B, Wong CN, Tong Y, Zhong JY, Zhong SSW, Wu WC, et al. TISIDB: an integrated repository portal for tumor–immune system interactions. Bioinformatics. 2019;35:4200–2.

7. Torre-Healy LA, Kawalerski RR, Oh K, Chrastecka L, Peng XL, Aguirre AJ, et al. Open-source curation of a pancreatic ductal adenocarcinoma gene expression analysis platform (pdacR) supports a two-subtype model. Commun Biol. 2023;6:163.

8. Tang Z, Kang B, Li C, Chen T, Zhang Z. GEPIA2: an enhanced web server for large-scale expression profiling and interactive analysis. Nucleic Acids Res. 2019;47:W556–60.

9. Han Y, Wang Y, Dong X, Sun D, Liu Z, Yue J, et al. TISCH2: expanded datasets and new tools for single-cell transcriptome analyses of the tumor microenvironment. Nucleic Acids Res. 2022;51:D1425–31.

10. Szklarczyk D, Franceschini A, Wyder S, Forslund K, Heller D, Huerta-Cepas J, et al. STRING v10: protein–protein interaction networks, integrated over the tree of life. Nucleic Acids Res. 2015;43:D447–52.

11. Karlsson M, Zhang C, Méar L, Zhong W, Digre A, Katona B, et al. A single–cell type transcriptomics map of human tissues. Sci Adv. 2021;7:eabh2169.

12. Cheung PF, Yang J, Fang R, Borgers A, Krengel K, Stoffel A, et al. Progranulin mediates immune evasion of pancreatic ductal adenocarcinoma through regulation of MHCI expression. Nat Commun. 2022;13:156.

13. Lahusen A, Minhöfer N, Lohse K-A, Blechner C, Lindenmayer J, Eiseler T, et al. Pancreatic cancer cell-intrinsic transglutaminase-2 promotes T cell suppression through microtubule-dependent secretion of immunosuppressive cytokines. J Immunother Cancer. 2025;13:e010579.

14. Monaco G, Chen H, Poidinger M, Chen J, Magalhães JP de, Larbi A. flowAI: automatic and interactive anomaly discerning tools for flow cytometry data. Bioinformatics. 2016;32:2473–80.

15. Aghaeepour N, Simonds EF, Knapp DJHF, Bruggner RV, Sachs K, Culos A, et al. GateFinder: projection-based gating strategy optimization for flow and mass cytometry. Bioinformatics. 2018;34:4131–3.

16. Becht E, Simoni Y, Coustan-Smith E, Evrard M, Cheng Y, Ng LG, et al. Reverse-engineering flow-cytometry gating strategies for phenotypic labelling and high-performance cell sorting. Bioinformatics. 2018;35:301–8.

17. Samusik N, Good Z, Spitzer MH, Davis KL, Nolan GP. Automated mapping of phenotype space with single-cell data. Nat Methods. 2016;13:493–6.

18. Gassen SV, Callebaut B, Helden MJV, Lambrecht BN, Demeester P, Dhaene T, et al. FlowSOM: Using self‐organizing maps for visualization and interpretation of cytometry data. Cytom Part A. 2015;87:636–45.

19. Barone SM, Paul AG, Muehling LM, Lannigan JA, Kwok WW, Turner RB, et al. Unsupervised machine learning reveals key immune cell subsets in COVID-19, rhinovirus infection, and cancer therapy. eLife. 2021;10:e64653.

20. Diggins KE, Greenplate AR, Leelatian N, Wogsland CE, Irish JM. Characterizing cell subsets using marker enrichment modeling. Nat Methods. 2017;14:275–8.

21. Livak KJ, Schmittgen TD. Analysis of Relative Gene Expression Data Using Real-Time Quantitative PCR and the 2−ΔΔC T Method. Methods. 2001;25:402–8.

22. Frank E, Hall MA, H. and I, Witten IH. Online Appendix for “Data Mining: Practical Machine Learning Tools and Techniques”, Morgan Kaufmann, Fourth Edition, 2016 [Internet]. The WEKA Workbench. Available from: <https://ml.cms.waikato.ac.nz/weka/Witten_et_al_2016_appendix.pdf>

23. Youden WJ. Index for rating diagnostic tests. Cancer. 1950;3:32–5.

24. Matthews BW. Comparison of the predicted and observed secondary structure of T4 phage lysozyme. Biochim Biophys Acta (BBA) - Protein Struct. 1975;405:442–51.

25. Blaauboer A, Koetsveld PM van, Mustafa DAM, Dumas J, Dogan F, Zwienen S van, et al. The Class I HDAC Inhibitor Valproic Acid Strongly Potentiates Gemcitabine Efficacy in Pancreatic Cancer by Immune System Activation. Biomedicines. 2022;10:517.

26. Danaher P, Warren S, Ong S, Elliott N, Cesano A, Ferree S. A gene expression assay for simultaneous measurement of microsatellite instability and anti-tumor immune activity. J Immunother Cancer. 2019;7:15.

27. Faul F, Erdfelder E, Buchner A, Lang A-G. Statistical power analyses using G*Power 3.1: Tests for correlation and regression analyses. Behav Res Methods. 2009;41:1149–60.
